# Atomic Cu‐N‐P‐C Active Complex with Integrated Oxidation and Chlorination for Improved Ethylene Oxychlorination

**DOI:** 10.1002/advs.202205635

**Published:** 2023-01-19

**Authors:** Hongfei Ma, Xiuhui Zheng, Hao Zhang, Guoyan Ma, Wei Zhang, Zheng Jiang, De Chen

**Affiliations:** ^1^ Department of Chemical Engineering Norwegian University of Science and Technology Sem sælands vei 4 Trondheim 7034 Norway; ^2^ State Key Laboratory of Heavy Oil Processing China University of Petroleum Qingdao Shandong 266580 P. R. China; ^3^ Institute of Functional Nano & Soft Materials Laboratory (FUNSOM) Jiangsu Key Laboratory for Carbon‐Based Functional Materials & Devices Joint International Research Laboratory of Carbon‐Based Functional Materials and Devices Soochow University Suzhou 215123 P. R. China; ^4^ Shanghai Synchrotron Radiation Facility Zhangjiang Lab Shanghai Advanced Research Institute Chinese Academy of Sciences Shanghai 201210 P. R. China; ^5^ College of Chemistry and Chemical Engineering Xi'an Shiyou University Xi'an Shaanxi 710065 P. R. China

**Keywords:** coordination, copper, ethylene oxychlorination, modulating, stability

## Abstract

Fine constructing the chemical environment of the central metal is vital in developing efficient single‐atom catalysts (SACs). Herein, the atomically dispersed Cu on the N‐doped carbon is modulated by introducing Cu—P moiety to Cu—N—C SAC. Through fine‐tuning with another heteroatom P, the Cu SAC shows the superior performance of ethylene oxychlorination. The Cu site activity of Cu‐NPC is four times higher than the P‐free Cu‐NC catalyst and 25 times higher than the Ce‐promoted CuCl_2_/Al_2_O_3_ catalyst in the long‐term test (>200 h). The selectivity of ethylene dichloride can be splendidly kept at ≈99%. Combined experimental and simulation studies provide a theoretical framework for the coordination of Cu, N, and P in the complex active center and its role in effectively catalyzing ethylene oxychlorination. It integrates the oxidation and chlorination reactions with superior catalytic performance and unrivaled ability of corrosive‐HCl resistance. The concept of fine constructing with another heteroatom is anticipated to provide with inspiration for rational catalyst design and expand the applications of carbon‐based SACs in heterogeneous catalysis.

## Graphical abstract

The atomically dispersed single‐atom Cu on N and P co‐doped porous carbon catalyst (Cu‐NPC) is synthesized using a facile one‐pot fabrication route. The Cu—N*_x_* single atom catalyst is fine‐tuned by another hetero atom P, forming a Cu–N–P–C complex center. It exhibits high activity, selectivity and stability toward ethylene oxychlorination, a very industrially relevant application.

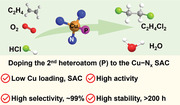

## 1 Introduction

Poly(vinyl chloride) (PVC) is one of the most widely used polymer materials in various fields.^[^ ^1^ ^]^ Based on the source (C_2_H_4_ or C_2_H_2_) used in the process, there are primarily two ways to generate vinyl chloride, the monomer of PVC, also known as VCM. The most important way to produce VCM is called a balanced VCM process via two steps.^[^ ^1c^ ^]^ Briefly, ethylene dichloride (EDC, C_2_H_4_Cl_2_) is first produced by ethylene chlorination or oxychlorination, followed by thermal cracking to produce VCM.^[^ ^1b^ ^]^ The HCl produced as the byproduct in the cracking step can be recycled into the oxychlorination process. Ethylene oxychlorination, a vital process to close the chlorine loop, is commonly used in western countries.^[^ ^1^, ^2^ ^]^ Due to the arising environmental issue, which has elevated the balanced VCM process to a leading‐edge way for VCM production.^[^ ^1b^ ^]^

In the oxychlorination process, C_2_H_4_, O_2_ and HCl react together over a CuCl_2_/*γ*‐Al_2_O_3_‐based catalyst, following a three‐step redox reaction cycle.^[^ ^1^, ^3^ ^]^ The reaction follows Mars–van Krevelen mechanism where CuCl partakes in the redox cycle. It is highly volatile at an operating temperature of roughly 250 °C in the industry.^[^ ^1b^ ^]^ Thus, Cu loss and aggregation are serious issues for the chemical plant, which gives rise to catalyst deactivation and byproduct formation, including CO*_x_* and other chlorides.^[^ ^1b^ ^]^ Besides, the catalytic performance is closely related to the reaction rate of the half‐reactions, such as reduction, oxidation and hydrochlorination. For instance, the catalyst with a slow oxidation rate will result in a high amount of CuCl formed on the catalyst, which lowers the catalyst's stability and selectivity.^[^ ^1b,c^ ^]^ Thus, a lot of efforts have been devoted to improving the stability of the CuCl_2_/Al_2_O_3_‐based catalyst. Alkali and alkali earth metals were used as promoters to enhance activity and stability.^[^ ^1b,c^ ^]^ Recently Perez‐Ramirez and his co‐workers developed other catalytic materials like CeO_2_,^[^ ^4^ ^]^ rare earth oxychloride,^[^ ^5^ ^]^ and iron phosphate^[^ ^6^ ^]^ to catalyze ethylene oxychlorination for EDC and/or VCM production with good stabilities, which helped us to move our eyes on some new catalytic materials for this industrially‐relevant reaction. Furthermore, it is imperative to address the product selectivity and reaction activity at lower reaction temperatures.

Single‐atom catalysts (SACs) have emerged as a new frontier in heterogeneous catalysis owing to the potential to maximize the metal utilization efficiency and the well‐defined structures.^[^ ^7^ ^]^ It has advanced very rapidly and led to promising catalyst developments, which can give high catalytic performance by using low metal loading. The chemical reactions are not necessarily driven by the central metal atom alone, but often jointly with the coordinated atoms.^[^ ^8^ ^]^ Among others, N‐doped carbon is one impressive support for SAC, due to the strong metal‐N covalent bond and N atoms providing anchoring sites for stabilizing the metal atom, which can further improve the catalyst stability.^[^ ^8^, ^9^ ^]^ On the other hand, the uniformly dispersed, strongly anchored, and typically electron‐deficient nature of SACs allows us to tune the structure in a controllable manner.^[^ ^10^ ^]^ Indeed, precisely constructing the structure has great potential to affect the reaction selectivity, as the reaction selectivity is closely correlated with the strength and configuration of the reactant, product adsorption or by changing the reaction pathway.^[^ ^10a^ ^]^ Nevertheless, SACs have not been explored in more challenging reactions involving both oxidant and HCl gases. Therefore, considering the above‐mentioned advantages, it would be interesting to assess the Cu catalyst with an atomically dispersed complex and investigate the potential for ethylene oxychlorination.

Herein, we report the atomically dispersed Cu SAC supported on N and P co‐doped porous carbon materials were prepared by a simple and easily scalable one‐pot synthesis method. The traditional Cu—N—C type catalyst was modified by doping another heteroatom of P in a carbon matrix. The atomically dispersed Cu‐NPC catalyst exhibited good catalytic performance with long‐term durability, EDC selectivity and activity, which is 4 times higher than the P‐free Cu‐NC catalyst. It outperformed the Ce‐promoted CuCl_2_/Al_2_O_3_‐based catalyst (≈25 times higher). To the best of our knowledge, this is the first time reporting that the carbon‐based Cu SAC shows high reactivity, and stability as well as EDC selectivity in ethylene oxychlorination, a very industrially relevant reaction. The detailed experimental and density functional theory (DFT) study provides a theoretical framework for the new catalytic reaction pathway. The outstanding catalytic performance benefits from the rational control of the coordination environment of the atomically dispersed Cu atoms.

## 2 Results

### 2.1 Preparation and Textural Properties of the Atomically Dispersed Cu Catalyst

M—N—C (M refers to metal) materials have been intensively investigated as a promising and typical type of single‐atom carrier and have been used in different catalytic reactions.^[^ ^11^ ^]^ Although a lot of methods have been reported for synthesizing this type of SACs, a method of simple and easy scale‐up is still highly needed to make this type of catalyst more attractive. Herein, the atomically dispersed Cu on the N and P co‐doped carbon catalysts were synthesized using lignin, which is the second most abundant renewable polymer and contributes a large part of the energy content of lignocellulosic biomass. N_2_ absorption–desorption results (Table S1, and Figure S1 and S2, Supporting Information) demonstrate a high specific surface area (above 2000 m^2^ g^−1^) of the N and P co‐doped carbon materials, which is reported that it can provide convenience for hosting and stabilizing single metal atoms.^[^ ^12^ ^]^ The pore size analysis (Figure S2, Supporting Information) shows the mesoporous structure on all carbon materials with the main peaks existing (≈2.8 nm), which is also confirmed by the isotherm curve in Figure S1, Supporting Information, showing a type‐I isotherm curve, the typical mesopore character. The high surface area and the porous structure can endow the Cu SACs catalyst with more accessible active sites and enhance the mass transfer for the reaction in the following part. The Cu loading was determined to be 0.24 wt% by the elemental analysis using inductively coupled plasma‐optical emission spectrometry (ICP‐OES), as shown in Table S2, Supporting Information. The N and P concentrations are 1.63% and 0.1%, respectively. The XRD patterns of all the samples of NPC, Cu‐NC and Cu‐NPC show no peaks of metallic or oxidic Cu species that can be observed, as shown in Figure S3, Supporting Information. The existence of the two broad peaks located at roughly 26° and 43° are assigned to the graphitic carbon.^[^ ^2^, ^13^ ^]^

### 2.2 Identification of the Atomic Dispersion of Cu

**Figure 1:**
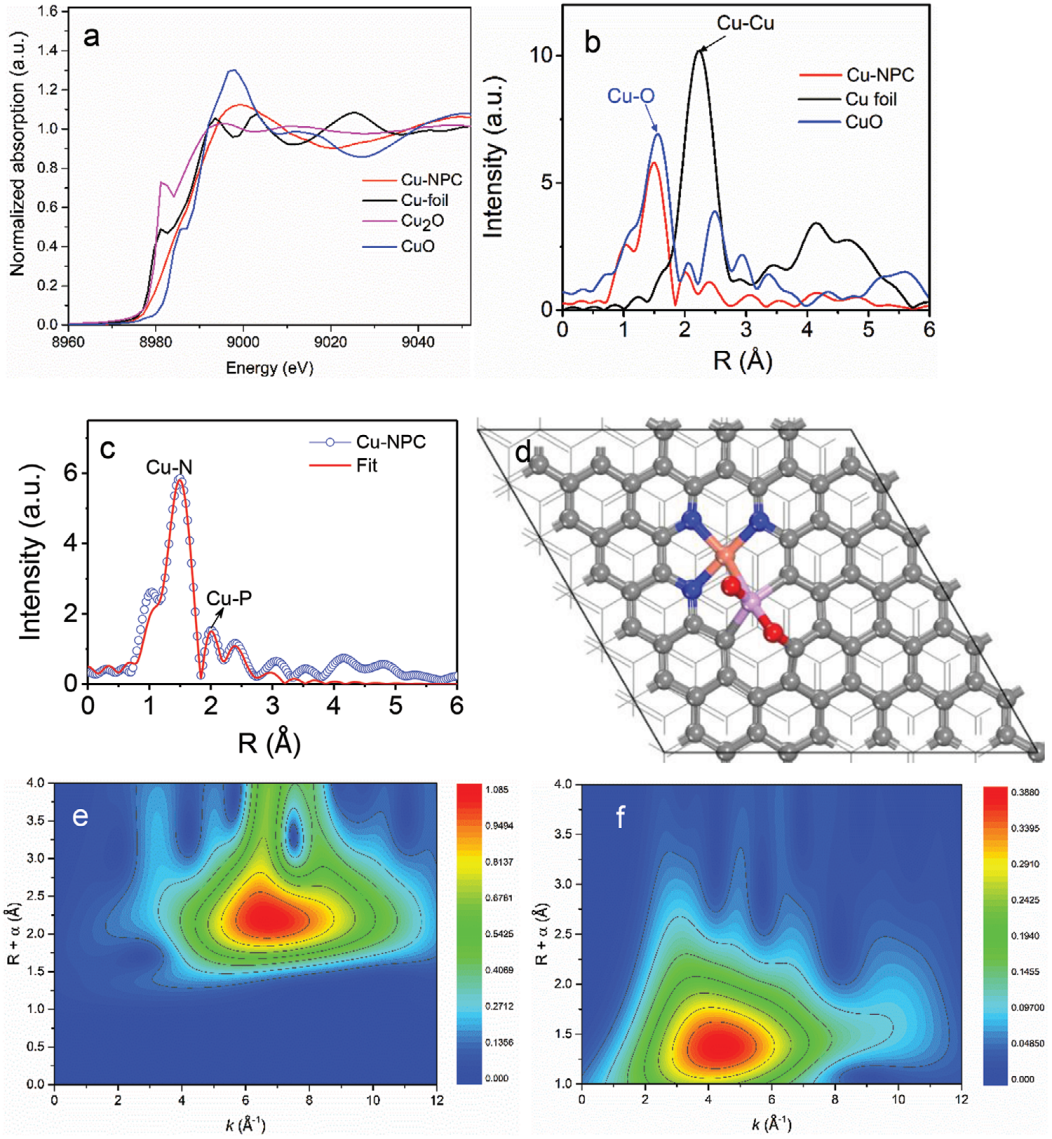
X‐ray absorption spectra of Cu‐NPC catalyst. a) Cu K‐edge XANES spectra of Cu‐NPC and the references. b) FT‐EXAFS spectra of Cu K‐edge for Cu‐NPC, Cu foil, and CuO. c) EXAFS spectra of the data and fitting curves at the Cu K‐edge. d) Proposed schematic model of Cu‐NPC (grey: carbon; red: oxygen; blue: nitrogen; the light shade of red: Cu; light magenta: phosphorus). Wavelet transformed plots of the Cu K‐edge EXAFS signal in e) Cu foil, and f) Cu‐NPC.

To verify the atomic dispersion of Cu and identify the Cu local coordination environment and modulation of the electronic structure on the N, P‐doped carbon, we performed the X‐ray absorption near‐edge structure (XANES) and extended X‐ray absorption fine structure (EXAFS) spectroscopy measurement. In the Cu K‐edge XANES spectra (**Figure** **1**a), both the adsorption edges and transition energies of Cu‐NPC are close to that of CuO, indicating that the oxidation state of Cu species in the Cu‐NPC is close to +2. The chemical valence state of the Cu on the Cu‐NPC was furtherly investigated by X‐ray photoelectron spectroscopy (XPS), as shown in Figure S4a, Supporting Information. The Cu 2p peak is located at ≈934.6 eV, which is close to the Cu^2+^,^[^ ^14^ ^]^ suggesting the chemical valence of the Cu on the N, P‐doped carbon is close to +2. Furthermore, the coordination of the atomically dispersed Cu is determined by the Fourier‐transformed (FT) EXAFS spectra of the Cu K‐edge analysis (Figure 1b). In contrast to the reference of Cu foil, the Cu‐NPC catalyst does not show any prominent peaks at the position of the Cu—Cu path, demonstrating the Cu species on the carbon surface is atomically dispersed,^[^ ^15^ ^]^ which also can be seen from the high angle annular dark‐field scanning transmission electron microscopy (HAADF‐STEM) shown in Figure S5, Supporting Information, excluding the existence of Cu nanoparticles. Herein, we should mention that due to the limitation of the detecting method, Cu dimers or trimers might also exist on the catalyst.^[^ ^7^, ^16^ ^]^ Based on the above findings, a fit to the EXAFS data for Cu‐NPC was performed, as shown in Figure 1c and Table S3, Supporting Information. The fitting curves exhibit a prominent peak at 1.9–2.0 Å derived from the first shell of the Cu—N scattering path with a coordination number of 3.5, plus a weak contribution at 2.4 Å originating from the Cu—P path with a coordination number of 0.4.^[^ ^17^ ^]^ Based on these findings, the proposed possible local structures of Cu‐NPC involve a coordination environment of one copper atom coordinated with nitrogen and phosphorus atoms, as shown in Figure S6, Supporting Information. The most stable structure is shown in Figure 1d.

Another solution to identify the local coordination environment is to use the wavelet transformed (WT) EXAFS, owing to the high sensitivity to the chemical nature of the scatters surrounding the absorbing atom,^[^ ^18^ ^]^ as shown in Figure 1e,f. Cu foil affords a lobe at (≈2.25 Å, 7 Å^−1^), which is attributed to Cu—Cu contribution. The absence of a lobe at a high *k‐*value in Cu‐NPC (Figure 1f) indicates the central Cu atom does not bind to heavy atoms (e.g., Cu). On the contrary, there exists a lobe at a lower *k*‐value (≈4 Å^−1^), which can be ascribed to Cu—N coordination,^[^ ^11^, ^19^ ^]^ and no Cu—Cu contributions are detected. It further demonstrates the atomic dispersion of the Cu atom in Cu‐NPC.

To further demonstrate the doping of N and P atoms, the high resolution of the N 1s XPS spectra of Cu‐NPC and NPC were analyzed. It revealed the presence of different N species: pyridinic N, pyrrolic N and graphitic N,^[^ ^9^, ^10^, ^13^ ^]^ as shown in Figures S4b and S7a, Supporting Information. However, the N 1s XPS spectra on the Cu‐NPC (shown in Figure S4b, Supporting Information) further feature a new bond (≈399.0 eV), indicating the formation of the Cu—N bond.^[^ ^9^, ^20^ ^]^ The doping of P to NPC and Cu‐NPC is also confirmed with P 2p XPS spectra, shown in Figures S4c and S7c, Supporting Information. The P 2p spectra showed a main peak at ≈134 eV, mainly referring to the P—O bonding on both Cu‐NPC and NPC catalysts, which can be denoted as PO*_x_*. The P—C bond located around 132 eV is absent, representing these P atoms are in a high oxidation state.^[^ ^21^ ^]^

As a comparison, the typical Cu‐NC catalyst was also prepared without adding P. The atomic dispersion of Cu on the Cu‐NC catalyst can also be evidenced by the absence of Cu—Cu scattering contribution, as shown in Figure S8, Supporting Information. Pyridinic N, pyrrolic N and graphitic N species are also observed on the N 1s XPS spectra as shown in Figure S9, Supporting Information. To further understand the structure changes by introducing P, Bader charge analysis was performed, as shown in Figure S10, Supporting Information. It suggests a lower oxidation state of Cu in Cu‐NPC (+0.71 e) than in Cu‐NC (+0.94 e). The difference is attributed to the doping of P, since P is losing electrons with positively charged status, which also verifies the interaction between Cu and P. Bader charge analysis shows that P‐doping can regulate the oxidation state of Cu atoms in Cu‐NPC, and thus the adsorption of reactants and products.

### 2.3 Catalytic Performance of Ethylene Oxychlorination

So far, we have designed and prepared the atomically dispersed Cu catalyst with the N, P‐doped carbon as the carrier. Stability is one key factor in heterogeneous catalysis, which is closely related to metal–support interaction, especially for SAC.^[^ ^7f^ ^]^ Ethylene oxychlorination, the most important industrial process for producing VCM, was chosen as the probe reaction to unveil the relationship between the stability and reactivity over the atomically dispersed Cu‐C‐based catalyst, and the complex and corrosive gas conditions can be good criteria for the stability evaluation.

**Figure 2:**
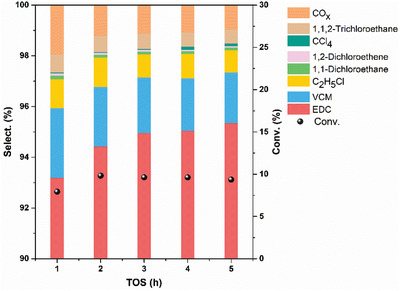
Conversion and product selectivity of the Cu‐NC catalyst without doping P. Reaction conditions: *W*_cat_ = 0.2 g, *T* = 250 °C, *P*_total_ = 1 bar, flow rate (mL min^−1^): C_2_H_4_/20%O_2_/20%HCl/N_2_: 4/10/40/4.

The typical Cu‐NC catalyst was first evaluated, and the results are shown in **Figure** **2**. Remarkable C_2_H_4_ conversion and EDC selectivity were obtained, and the EDC selectivity is more or less the same as the current mainly used CuCl_2_/Al_2_O_3_‐based catalysts.^[^ ^1b^ ^]^ However, other byproducts, chlorides and CO*_x_* are also formed, which are mainly reported as that they are produced from the EDC secondary reactions.^[^ ^1b,c^ ^]^ One main byproduct of the Cu‐NC catalyst, VCM, is mainly produced from EDC cracking, and the N‐doped carbon was also reported to be a good catalyst for this catalytic cracking reaction.^[^ ^2^, ^22^ ^]^ CO*_x_* formation, one of the main drawbacks of the carbon‐based catalyst, also occurred here, and it has been reported that the O‐species on the carbon surface are quite active and these species can react with the hydrocarbons, etc.^[^ ^23^ ^]^

**Figure 3:**
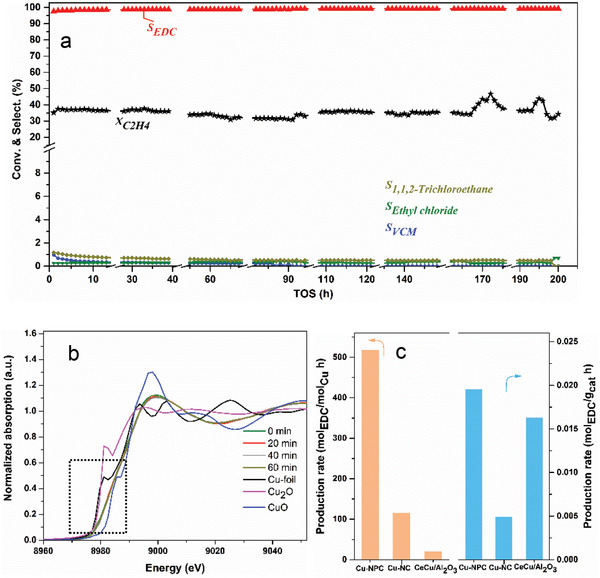
Ethylene oxychlorination catalytic performance. a) C_2_H_4_ conversion and product selectivity of Cu‐NPC catalyst. b) Cu K‐edge XANES spectra of Cu‐NPC during the reaction (performed at the same partial pressure with (a), but with a total flow rate of 10 mL min^−1^ in the capillary reactor. c) Comparison of the production rates (calculated from the reaction at the 3rd hour). Reaction conditions: *W*_cat_ = 0.2 g, T = 250 °C, *P*_total_ = 1 bar, flow rate (mL min^−1^): C_2_H_4_/20%O_2_/20%HCl/N_2_: 4/10/40/4. Note: the reaction was maintained during the breaking.

The intrinsic activity is usually determined by the local coordination environment of the atomically dispersed Cu. To check the influences caused by the local coordination environment of the Cu on the catalytic performance, the traditional Cu‐NC (N‐doped carbon) type catalyst was further modified by doping P (same procedures as the Cu‐NC catalyst, while adding H_3_PO_4_ during the synthesis) and forming the Cu—P bond. The catalytic results of the Cu‐NPC catalyst including the activity and product distributions are shown in **Figure** **3**. The Cu‐NPC catalyst shows high activity and selectivity during the 200 h on stream with the EDC selectivity of ≈99%. It is affording a 4‐times conversion than that of the Cu‐NC catalyst as shown in Figure 2. Both the C_2_H_4_ conversion and EDC selectivity are greatly enhanced and can be maintained during the 200‐h evaluation. By introducing the acidic P‐site, part of the basic N‐species can be neutralized, as shown by the HCl‐TPD in Figure S11, Supporting Information, that the adsorption of HCl on Cu‐NC is much stronger than that on Cu‐NPC, indicating the basic sites were partially neutralized as introducing P into the catalyst. Thus, making the EDC cracking reaction significantly weakened. Besides, the adsorption of EDC on Cu‐NPC is weaker than that on Cu‐NC catalyst, as shown in Figure S12, Supporting Information. Both the adsorption capacity and strength of EDC on Cu‐NC are much higher than Cu‐NPC. As soon as EDC* is formed, it would be easily desorbed to the gas phase. As for the high activity compared to the Cu‐NC catalyst, from the simulated EXAFS results we know, that the active center changed to Cu–N–P–C from Cu—N—C when doping P into the catalyst. Introducing Cu—P moiety into the Cu‐NC catalyst makes it more active and selective toward ethylene oxychlorination. And this complex active center is supposed to influence the whole reaction. Additionally, we will use experimental mechanism analysis and DFT simulation to systematically discuss this intrinsic explanation.

To get the real oxidation state of Cu under the real reaction conditions, we also performed the in situ XAS under the reaction atmosphere, as shown in Figure 3b. We can see the Cu K‐edge was located between Cu^+^ and Cu^2+^, but show a similar pattern with CuO, indicating the Cu oxidation state is close to Cu^2+^. The catalysts after the reaction were also characterized. No structure changes can be observed on XRD patterns and BET surface area, pore volume, and pore size compared to the fresh catalyst (Figure S13 and Table S1, Supporting Information). Also, no Cu loss can be observed as indicated by the ICP on the used catalyst after the reaction. Especially, the oxidation state of Cu can be maintained, as it is shown in Figure S14, Supporting Information, that no binding energy shift was observed. The catalyst structure after reaction was further studied by the EXAFS spectra, as shown in Figure S15, Supporting Information, that no peak characteristic for Cu—Cu bonds were detected on the samples after a 1‐h reaction, indicating no Cu growth or aggregation occurred during the reaction process, and the Cu coordination kept unchanged.

And the blank experiment on the NPC catalyst without adding Cu was also employed to verify the important role of atomically dispersed Cu in catalyzing ethylene oxychlorination to EDC, and the results are shown in Table S4, Supporting Information. CO_2_ was produced with the highest selectivity, then followed by VCM and EDC, and other chlorides at a rather low conversion (≈2%). To the best of our knowledge, this is the first time reporting the Cu SAC in this industrial process with imperial catalytic performance. As we know, CO_2_ is one of the important by‐products even for ethylene oxychlorination industrial catalysts. Herein, no CO_2_ was detected on this Cu‐NPC catalyst along with the 200‐h lifetime test.

The Ce‐doped CuCl_2_/Al_2_O_3_ was proved to be a highly active and stable catalyst for ethylene oxychlorination.^[^ ^1b^ ^]^ The catalytic performance on Ce‐doped CuCl_2_/Al_2_O_3_ catalyst had high EDC selectivity (≈99%) and C_2_H_4_ conversion, as shown in Figure S16, Supporting Information. Although a rather high EDC selectivity can be obtained, a gradually decreasing tendency of C_2_H_4_ conversion is occurring. Indicating the deactivation also occurs on the Ce‐promoted CuCl_2_/Al_2_O_3_ catalyst. While, the deactivation is more serious on the neat CuCl_2_/Al_2_O_3_ catalyst, as shown in Figure S17, Supporting Information, both the conversion and EDC selectivity are decreasing, especially the byproduct ethyl chloride, which increased to 13% after the 7‐h run. It indicates that adding promoters can improve the EDC selectivity, but not the deactivation of the CuCl_2_/Al_2_O_3_‐based catalysts. The production rate of Cu‐NPC is much higher (≈25 times) than the Ce‐doped CuCl_2_/Al_2_O_3_ catalyst, as shown in Figure 3c. Such a high intrinsic site activity made the catalyst highly active so that the production rate per catalyst mass was also higher compared to the CeCu/Al_2_O_3_ catalyst (≈1.2 times), as shown in Figure 3c and other CuCl_2_/Al_2_O_3_‐based catalyst reported in the literature as shown in Table S5, Supporting Information. However, the Cu‐NC shows a much lower activity per gram catalyst compared to the CeCu/Al_2_O_3_ catalyst, but a much higher activity in terms of the Cu amount. Herein, doping P into the Cu‐NC can greatly enhance the reactivity, in terms of per unit gram of catalyst and per unit amount of Cu. The results demonstrated that the Cu‐NPC is more active, selective, and stable for ethylene oxychlorination compared to the Ce‐doped CuCl_2_/Al_2_O_3_‐based catalysts.

### 2.4 Mechanism Study

The atomically dispersed Cu‐NPC catalyst has demonstrated superior catalytic performance for ethylene oxychlorination with outstanding stability and production rate compared to the typical CuCl_2_/Al_2_O_3_‐based catalyst. Detailed kinetic and mechanism studies using multiple temperature‐programmed surface reactions (TPSR) combined with DFT simulation were performed to elucidate the reaction pathway and provide insights into the coordinated Cu active sites.

**Figure 4:**
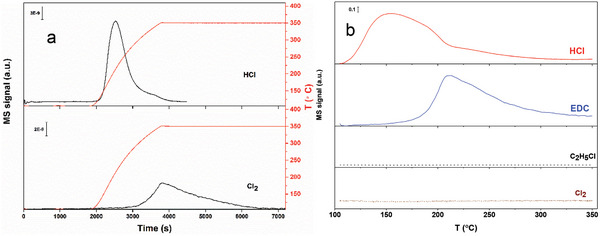
TPSR profiles of the Cu‐NPC catalyst (saturated adsorbed HCl). a) O_2_‐TPSR, 10% O_2_/Ar 100 mL min^−1^. b) C_2_H_4_‐TPSR, 10% C_2_H_4_/Ar 100 mL min^−1^. Conditions: *W*_cat_ = 0.2 g, ramping rate 10 °C min^−1^.

First, the HCl adsorption and desorption on the catalyst with and without Cu dispersed on NPC were performed, as shown in Figure S18, Supporting Information. It can rationalize that HCl is possibly mainly adsorbed on the N‐doped carbon surface, possibly not directly bonding with the Cu atom. It is also confirmed with the previous reports that HCl can be easily adsorbed on the N‐doped carbon, forming a N—H—Cl group.^[^ ^2^, ^24^ ^]^ The slightly lower capacity on Cu‐NPC is possibly due to the coordination of Cu with N sites. A TPSR of the oxidation of the pre‐adsorbed HCl was carried out on Cu‐NPC, Cu‐NC, and NPC (**Figure** **4**a and Figure S19, Supporting Information). The pre‐adsorbed HCl was desorbed at relatively low temperatures, and Cl_2_ formation was observed on Cu catalysts following the Deacon reaction process. However, no Cl_2_ gas was detected on the NPC catalyst. And it has been reported that the Cu‐based catalyst was the conventional catalyst for the high‐temperature Deacon reaction.^[^ ^25^ ^]^ The results suggested oxidative dissociation of adsorbed HCl to surface Cl* and OH*, which are important surface intermediates for EDC and H_2_O formation in ethylene oxychlorination. In addition, the factors of the lower peak temperature and smaller peak areas on Cu‐NPC compared to Cu‐NC revealed that coordination of P into the active sites weakened HCl adsorption.

However, the phenomenon of C_2_H_4_ desorption (Figure S20, Supporting Information) is opposite to that of HCl‐TPD. C_2_H_4_ adsorbs rather weakly on NPC and desorbs almost completely at a temperature lower than 200 °C. On Cu‐NPC, in addition to the weak adsorption peak, a relatively strong adsorbed C_2_H_4_ is observed, which desorbs at higher than 200 °C, close to the reaction temperature. It suggests that the atomically dispersed Cu atoms enhanced C_2_H_4_ adsorption at the reaction conditions, thus the reaction.

Another TPSR was performed to elucidate the reactivity of the surface Cl* and C_2_H_4_ on the two catalysts where the catalyst surfaces were first saturated with adsorbed HCl, followed by the introduction of C_2_H_4_ and O_2_ into the catalyst. As shown in Figure S21, Supporting Information, EDC is the main product, and almost no Cl_2_ and C_2_H_5_Cl were detected. It suggests that C_2_H_4_ can effectively attract surface Cl* toward the EDC formation, in which Cl* is formed by the oxidative dissociation of HCl with oxygen. It seems that the Cl additive reaction with C_2_H_4_ has a lower energy barrier than the Cl—Cl combination reaction, leading to a highly selective EDC formation. In addition, no C_2_H_5_Cl formation was detected suggesting a full oxidative dissociation of HCl, and no H* on the surface is available. The peak maximum temperature on the Cu‐NPC is lower than the one on the Cu‐NC catalyst, revealing a higher activity of Cu‐NPC, which is consistent with a higher activity of Cu‐NPC than the Cu‐NC at the steady state. In addition, no CO_2_ can be detected during the TPSR ramping, as shown in Figure S21b, Supporting Information (till the final temperature of 350 °C), it proved once again that the Cu‐NPC catalyst itself can be kept stable in ethylene oxychlorination, and it will not be self‐oxidized by the O_2_. In addition, the maximum peak temperature of EDC formation of the TPSR in the presence of gas‐phase O_2_ is 8 °C smaller on Cu‐NPC than on Cu‐NC, indicating enhanced oxygen activation on Cu‐NPC, most likely due to the presence of P that is highly oxyphilic.

As it is commonly reported that the oxygen‐containing functional groups are unavoidably introduced during the synthesis process.^[^ ^2^, ^23^ ^]^ A large amount of research has reported that O_2_ can be easily adsorbed on the N‐doped carbon surface,^[^ ^2^, ^23^ ^]^ which can also be seen from the O 1s XPS, shown in Figure S4d, Supporting Information. The C_2_H_4_‐TPSR without the gas‐phase O_2_ was performed on NPC, Cu‐NPC and Cu‐NC samples pre‐adsorbed HCl, respectively, to elucidate the role of the surface oxygen groups. The evolution of the formed species is shown in Figure 4b and Figure S22, Supporting Information. Like the TPSR in the presence of gas‐phase O_2_, EDC is the main product. It evidences that surface O‐species are highly active and can activate the HCl* to form Cl*. Almost no or a small amount of EDC was produced on NPC, indicating that the Cu active site catalyzes either the reaction of C_2_H_4_* and Cl* or/and oxidative HCl dissociation. More interestingly, the maximum peak temperature is much lower than that of the Cu‐NPC catalyst with the C_2_H_4_/O_2_‐TPSR shown in Figure S21a, Supporting Information. The reason can be ascribed that the adsorbed oxygen species on the carbon surface are highly active, and it was also commonly reported in the literature that they can participate in a variety of reactions.^[^ ^23^, ^26^ ^]^ Herein, it can directly join the reaction of oxidative dissociation of HCl*. While this kind of oxygen species is commonly reported as one main reason for CO*_x_* formation in alkane conversion reactions.^[^ ^23a,b^ ^]^ It has been reported that the selective blockage of the surface O‐groups on the carbon surface can prohibit the formation of CO*_x_*.^[^ ^2^, ^23^ ^]^ No CO*_x_* was detected on the Cu‐NPC catalyst, suggesting P doping partially blocked the surface oxygen sites. Another reason is the adsorbed O‐groups might selectively participate in the reaction to activate the HCl*, but not with C_2_H_4_ to produce CO*_x_*.

**Figure 5:**
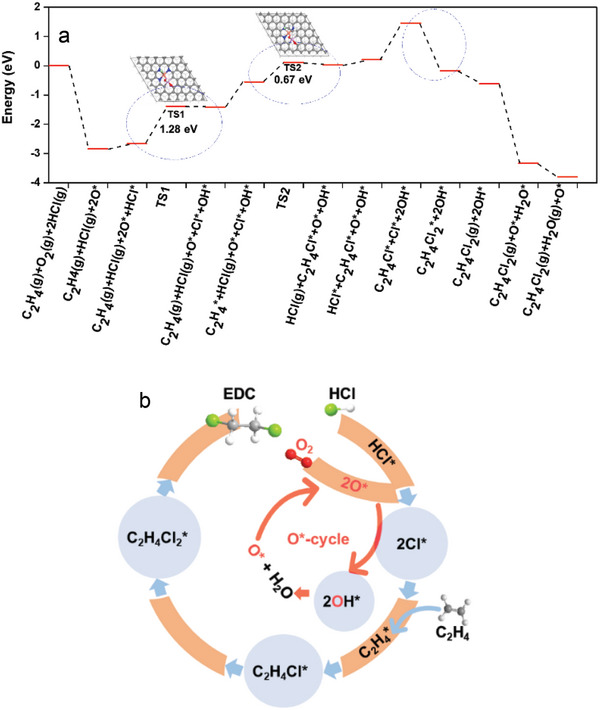
The reaction pathway for EDC production. a) Gibbs free energy diagram over the Cu‐NPC catalyst. b) Illustration of the reaction pathway for EDC production.

Based on the above discussion, the reaction mechanism in terms of the elementary steps is proposed (Table S6, Supporting Information) and was validated by the DFT study. And the imaginary frequencies of each transition state are also listed in Tables S7 and S8, Supporting Information. It involves the adsorption of C_2_H_4_, HCl and O_2_, oxidative dissociation of HCl*, the surface reaction of adsorbed C_2_H_4_* and Cl* to form C_2_H_4_Cl*, and the reaction of C_2_H_4_Cl* with Cl* to form EDC* on the surface, and EDC desorption. The Gibbs free energy profile is plotted with the reaction coordinate in **Figure** **5**a. Due to the strong oxyphilic of P with oxygen, O is strongly adsorbed and activated instantly on P sites (Figure S23, Supporting Information). The HCl adsorbed relatively weakly on the top of the N and Cu sites, where Cl is toward N and H is toward O. The transition state of HCl oxidative activation is close to the final state, where OH forms on the P site, and Cl* adsorbed on the top of N and Cu sites. The relatively weak adsorption of HCl resulted in a high energy barrier of oxidative dissociation of HCl.

In addition, OH*‐assisted HCl* activation was also checked out. The energy barrier (1.98 eV) is found to be much higher than the oxidative dissociation (1.28 eV), as shown in Figure S24, Supporting Information. Furthermore, the charge distribution of the HCl* adsorption on both O* and OH* was analyzed through the charge density difference and the Bader charge, as shown in Figure S25, Supporting Information. The charge accumulation is depicted in the yellow region and the charge depletion in the blue region. It indicates that the adsorption of HCl* on the O* site is much stronger than that on OH*, reflected by the adsorption energies of −0.58 and −0.29 eV on O* and OH*, respectively. It suggests that O* is the main active site to dissociate HCl*. The C_2_H_4_ is weakly adsorbed on the Cu site via *π* bond (Figure S26, Supporting Information), which reacts with adsorbed Cl* on N sites to form C_2_H_4_Cl* with a relatively low energy barrier (0.67 eV). The second step of chlorination occurs instantly without any energy barrier to form C_2_H_4_Cl_2_*. Cl* is preferred to react with C_2_H_4_, rather than self‐binding together to Cl_2_ as a much higher energy barrier is needed for the latter process.^[^ ^27^ ^]^ It seems EDC desorption is a rather easy process without energy barriers from the Gibbs energy profile, as it can also be reflected from the EDC‐TPD shown in Figure S12, Supporting Information, that the desorption of EDC occurs at a rather lower temperature range. And the weak adsorption is beneficial for the higher EDC selectivity, as it can be easily desorbed to the gas phase. Figure 5a displayed the calculated Gibbs free energy diagram of ethylene oxychlorination based on the above‐discussed reaction mechanism. From the Gibbs free energy profile, the oxidative HCl dissociation is the rate determine step of the whole reaction steps.

Together with transient kinetic results, in situ XAS and DFT calculation suggest a Cu–N–P–C active cluster with atomically precise multiple active sites, where atomically dispersed Cu coordinates with N and P in a carbon matrix (Cu‐NPC), similar to molecular ligand‐stabilized transition metal nanoclusters. Unlike the conventional CuCl_2_/Al_2_O_3_ catalyst catalyzing the ethylene oxychlorination reaction through three sequence steps, reduction, oxidation and hydrochlorination, CuCl_2_, CuCl and Cu_2_OCl_2_ compete on the Cu site. Multiple active sites of Cu, N, P, and O coordinated in the active cluster catalyze different elementary steps non‐competitively. Cu site enhances C_2_H_4_ adsorption and attraction of surface Cl*, N site enhances Cl adsorption, and P—O enhances oxygen activation. The active cluster stabilizes the transition state of the surface reaction, and lowers the energy barrier, resulting in an about 25 times higher activity than the Ce‐promoted CuCl_2_/Al_2_O_3_ catalyst.

The transient experiment and the in situ XAS were also performed, which revealed an evolution of the Cu oxidation state during the reaction and confirmed that the Cu is undergoing a redox cycle. When introducing C_2_H_4_ to the catalyst (Figure S27, Supporting Information), the Cu would be reduced from +2 to a lower oxidation state. While introducing HCl and O_2_ to the catalyst (Figure S28, Supporting Information), Cu can be re‐oxidized to Cu^2+^ again. At the steady state, the Cu keeps a high oxidation state during the reaction, possibly because of multifunctional active sites. In addition, coordinating the active site with P tunes the acidity that reduced the HCl adsorption strength and the secondary reaction of EDC. Moreover, by co‐doping N and P to the catalyst, the over‐oxidation and EDC secondary reaction to other chlorides can be well prohibited, and no CO*_x_* was produced in the ethylene oxychlorination reaction. So, tunning the Cu coordination by partially coordinating to P through the Cu—P pathway, the intriguing catalytic performance can be obtained via precise control of the atomically dispersed Cu‐NPC catalyst. The above‐discussed steps are illustrated in Figure 5b for an overview of the reaction mechanism. The synergetic effects of the different sites in the active complex make the atomically dispersed Cu‐NPC highly active, selective, and stable. It, therefore, rationalizes that Cu‐NPC leads to superior catalytic performance compared to Cu‐NC and NPC catalysts.

## 3 Conclusion

In summary, the atomically dispersed Cu on carbon support with tailored local coordination with N and P was proposed by a simple one‐pot synthesis method. By introducing second heteroatoms of P into the typical Cu‐NC catalyst, both the activity and EDC selectivity were greatly enhanced in the 200‐h evaluation. The in situ XAS and DFT study provides a comprehensive framework for the active cluster with multiple active sites (Cu–N–P–C) as the active center to effectively catalyze ethylene oxychlorination. The atomically dispersed Cu catalyst (Cu‐NPC) with coordination of P and N shows high activity, selectivity and stability, which could inspire the next‐generation industrial application of ethylene oxychlorination. A new reaction mechanism was proposed, following the oxidative dissociation of HCl with the assistance of oxygen, with Cl* produced and chlorinating C_2_H_4_ to EDC on the complex active sites. This work provides a novel and efficient catalyst for ethylene oxychlorination and paves a new versatile method to fabricate other active complex catalysts with transition metal single atoms. Additionally, the conventional Cu—N*_x_* type catalyst can be modified by other dopants for better catalytic performance, which also demonstrates the significance of constructing well SAC for use in heterogeneous catalysis with high stability in future research. It has the potential to provide valuable guidance toward the rational design of highly selective and robust SACs for other demanding catalytic applications.

## 4 Experimental Section

Full experimental procedures are provided in the Supporting Information.

## Conflict of Interest

The authors declare no conflict of interest.

## Author Contributions

H.M. performed the catalyst preparation and characterization and catalytic measurements, and wrote the manuscript. X.Z. did the DFT calculation. H.Z. and Z.J. helped with the analysis of XAS data. G.M. did the XPS measurements. W.Z. helped with the in situ XAS characterization and discussion. D.C. guided the project and funding acquisition. All authors contributed to the discussion of the manuscript.

## Supporting information

Supporting Information

## Data Availability

The data that support the findings of this study are available from the corresponding author upon reasonable request.
