# Supplementary material for: Atomic Cu‐N‐P‐C Active Complex with Integrated Oxidation and Chlorination for Improved Ethylene Oxychlorination
Source: Adv Sci (Weinh). 2023 Jan 19;10(8):2205635. doi: 10.1002/advs.202205635 (PMC10015856; doi:10.1002/advs.202205635)
Supplement: Supplementary file 1 — Supporting Information [file ADVS-10-2205635-s001.pdf]

ADVANCED SCIENCE
Open Access

Supporting Information

for *Adv. Sci.*, DOI 10.1002/advs.202205635

Atomic Cu-N-P-C Active Complex with Integrated Oxidation and Chlorination for Improved Ethylene Oxychlorination

*Hongfei Ma, Xiuhui Zheng, Hao Zhang, Guoyan Ma, Wei Zhang, Zheng Jiang and De Chen**

Atomic Cu-N-P-C Active Complex with Integrated Oxidation and Chlorination for Improved Ethylene Oxychlorination

*Hongfei Ma, Xiuhui Zheng, Hao Zhang, Guoyan Ma, Wei Zhang, Zheng Jiang, and De Chen**

*Corresponding Author: de.chen@ntnu.no

Content list:

S1. Experimental methods

S2. Results and discussions

Figure S1−S28

Table S1−S8

S1. Experimental methods

1. Synthesis of single atom Cu catalyst

The Cu single atom catalyst, co-doped with nitrogen and phosphorus, was prepared by an impregnation and pyrolysis method. Lignin (purchased from St1 Renewable Energy) was used as the carbon source and support. Typically, a 400 ml solution was prepared, containing 1.5 mol/L H_3PO_4 (Sigma-Aldrich) and 1.78 mmol/L $Cu(NO_3)_2$ (Sigma-Aldrich). 6 g of lignin was dispersed in a 200 ml precursor solution, and stirred for 30 min at room temperature. The obtained suspension was filtered with the rest 200 ml solution, on a vacuum filtration and the filter cake was dried at room temperature overnight. The dried samples were then loaded into a fixed bed reactor, heated up to 1000 °C in Ar at the rate of 5 °C/min, and kept at 1000 °C for 2h in NH_3 with the flow rate of 50 ml/min. The obtained black powders were refluxed in distilled water at 110 °C overnight to remove any impurities like soluble salts. Then, the samples were washed and drought at 80 °C. The obtained sample was the Cu single atom catalyst, denoted as the Cu-NPC. The elemental analysis of Cu and P was performed by using inductively coupled plasma optical emission spectrometry (ICP-OES). For comparison, the same procedure was performed on the lignin without adding $Cu(NO_3)_2$ during the synthesis process.

The atomically dispersed Cu catalyst without P (denoted as Cu-NC) is prepared following the same procedure without adding H_3PO_4 to the solution. And the catalyst without adding $Cu(NO_3)_2$ precursor but followed by the same calcination procedures is denoted as NPC.

The alumina-supported $CuCl_2$ catalysts were prepared by the incipient wetness impregnation method. ($CuCl_2 \cdot 2H_2O$ and/or $CeCl_3$, Sigma-Aldrich, ≥99%) were co-impregnated on the γ-Al_2O_3 (Puralox SCCA-30/170) with the calculated percentages, 5 wt% Cu, and the molar ratio of Ce-to-Cu is 0.4 (with the 4.4 wt% of Ce). After impregnation, the samples were put into the oven at 25 °C for 10 h, after that, the samples were heated to 120 °C with a ramping rate of 2 °C/min and kept for 6 h.

2. Catalytic performance evaluation

The ethylene oxychlorination was performed on a fixed bed reactor to evaluate the performance of the single atom catalyst. The catalyst (0.2 g) was heated to the target temperature of 250 °C in Ar with a flow rate of 50 ml/min. The reactant gases (AGA AS) (C_2H_4/20%O_2/20%HCl/N_2: 4/10/40/4 ml/min, with stoichiometric ratios of 2:1:4) were

introduced into the reactor by mass flow controllers. The effluent gas compositions were analyzed by an online GC (Agilent 7890B) equipped with a thermal conductivity detector (TCD) and a flame ionization detector (FID). N_2 is used as the internal standard, the products including all the chlorides are analyzed on FID, and the CO_x is analyzed on TCD.

The C_2H_4 conversion and product selectivity were calculated with the following equations, where C_i denotes the molar fraction of a product *i*. the vi means the stoichiometric parameter for the converting of C_2H_4 to the product *i* to the number of carbon atom numbers, for example, $C_2H_4 + 3O_2 \rightarrow 2CO_2 + 2H_2O$, v_i=2.

$$X_{C_2H_4} = \frac{C_2H_{4_{inlet}} - C_2H_{4_{outlet}}}{C_2H_{4_{inlet}}} \times 100\%$$

$$S_i = \frac{C_i}{\sum C_i/v_i} \times 100\%$$

The production rate of EDC was calculated for comparison of the two catalysts, the following equations are used, one is based on the gram of the catalyst, and the other one is based on the molar of Cu.

$$production\ rate\ (mol_{EDC} \cdot g_{cat}^{-1} \cdot h^{-1}) = \frac{F_{Ethylene} \times 60 \times Conv. \times Select.}{22400 \times w_{cat}}$$

$$production\ rate(mol_{EDC} \cdot mol_{Cu}^{-1} \cdot h^{-1}) = \frac{F_{Ethylene} \times 60 \times Conv. \times Select.}{22400 \times w_{cat} \times wt\%/M}$$

where, $F_{Ethylene}$ is the flow rate of C_2H_4 (ml/min), *Conv.* is the conversion of C_2H_4, *Select.* is the selectivity of EDC, w_{cat} is the mass of catalyst, *wt%* is the Cu loading, *M* is the molar mass of copper. Herein, the conversion at the 3rd hour was used for the calculation.

3. Characterizations

3.1 Physical structure of the catalysts

The Brunauer Emmett-Teller (BET) analyses were performed on a Tristar II 3020 (Micromeritics Instrument) using N_2 adsorption at 77 K. The samples were evacuated at 100 °C overnight before measurement. The pore size distribution and pore volume were calculated based on the Density Functional Theory (DFT) model method. X-ray diffraction (XRD) was performed using a Bruker D8 Advance DaVinci with Cu Kα radiation at the wavelength of 0.154 nm.

HAADF-STEM was carried out on the JEOL JEM-ARM200F integrated with CEOS spherical aberration corrections in the image and probe-forming lenses at 200 kV. The samples were prepared by dispersing a small amount of catalyst in isopropanol, and the solution was placed in an ultrasonic bath. A droplet of the solution was then transferred to a gold mesh TEM grid covered by holey carbon.

3.2 X-ray photoelectron spectroscopy

X-ray photoelectron spectroscopy (XPS) was performed on a Thermo Fisher K-alpha X-ray photoelectron spectrometer system, equipped with monochromator Al Kα radiation, for the surface and chemical valence state analysis. The C 1s peak at 284.8 eV was used as a calibration for other elements.

3.3 X-ray absorption spectroscopy (XAS) analysis

The X-ray absorption spectroscopy including X-ray absorption near-edge spectra (XANES) and extended X-ray absorption fine structure (EXAFS) of Cu K-edge were collected at BL 14W1 beamline station in Shanghai Synchrotron Radiation Facility (SSRF), using the fluorescence mode by a fixed-exit double crystal Si (111) monochromator at ambient air. The electron storage ring in SSRF was operated at 3.5 GeV, with a maximum current of 250 mA. The Cu foil was used as the reference to conduct the energy calibrations. The operando XAS of Cu K-edge were collected at the Swiss-Norwegian beamline BM 31 at the ESRF, using the fluorescence model by Si (111) and Si (311) monochromator crystals.

3.4 HCl temperature-programmed desorption (HCl-TPD)

HCl-TPD was carried out at the fixed bed reactor with an online MS (Hiden Analytical HPR-20 R&D) recording the effluent gases. The samples (0.2 g) were heated to 100 °C in Ar and kept for 1 h to purge out the moisture. When the samples were cooling down to room temperature, HCl (20% in Ar) was subsequently introduced into the reactor with a flow rate of 50 ml/min for 1 h for saturated adsorption. The samples were then heated to 100 °C and kept for another 1 h to purge out the weakly adsorbed HCl. Once a stable MS baseline was obtained, the desorption was initiated by increasing the temperature to 350 °C with a ramp rate of 10 °C/min while recording the effluent gases on the online MS.

3.5 Ethylene temperature-programmed desorption (C_2H_4-TPD)

The samples (0.2 g) were firstly treated at 100 °C in Ar for 1 h to purge out the moisture and cool down to 30 °C. The samples were then saturated with 20% C_2H_4/Ar for 1 h. Subsequently, the samples were purged with Ar at 100 °C to remove the physical adsorbed C_2H_4 with a flow rate of 100 ml/min. Once a stable MS (Hiden Analytical HPR-20 R&D) baseline was obtained, chemisorbed C_2H_4 was desorbed by heating from 100 °C to 350 °C with a rate of 10 °C/min with the effluent gas monitored by an online MS. The final temperature was kept for 30 min until a stable signal was obtained.

3.6 temperature-programmed surface reactions (TPSR)

The temperature-programmed surface reactions were performed on the homemade setups with the fixed bed reactor combing with online mass spectrometry (MS, Hidden Analytic HPR-20 R& D). The samples (0.2 g) were heated to 100 °C in Ar and kept for 1 h to remove the moisture in a fixed bed reactor. The samples were then cooled down to room temperature, and HCl (100 ml/min, 20% in Ar) was introduced into the reactor for 1 h to adsorbed saturate at room temperature. Subsequently, it was heated to 100 °C and kept for 1 h to purge out the physical adsorbed HCl in Ar with a flow rate of 100 ml/min. When a stable MS baseline was obtained, O_2 (10% in Ar) was introduced into the reactor with a flow rate of 100 ml/min, while the samples were heated to 350 °C with a ramp rate of 10 °C/min. The effluent gases were recorded by an online MS for analysis, and it will be named O_2-TPSR in the present work.

Another TPSR with flowing C_2H_4 and O_2 simultaneously into the catalyst surface after it was saturated with adsorbed HCl was performed in similar reaction conditions (with the fresh catalysts), and it will be named C_2H_4/O_2-TPSR.

Similar procedures as above of the C_2H_4/O_2-TPSR were performed on the fresh catalyst, except only C_2H_4 was introduced without O_2, it will be named C_2H_4-TPSR hereafter.

3.7 Ethylene dichloride temperature programmed desorption (EDC-TPD)

The EDC-TPD was also performed on the homemade setup with the fixed-bed reactor. The catalysts (0.2 g) were pretreated at 100 °C in the Ar atmosphere for 1 hour to purge out the adsorbed moisture. Then the samples were cooled down to room temperature. EDC was introduced by bubbling using Ar as the carrier gas flowing through the catalyst at room temperature for 1 hour. After adsorption, the samples were heated to 100 °C in Ar to purge out the physical and/or weakly adsorbed EDC for 1 hour. Then the TPD was performed by

heating the catalyst to 350 °C at a ramping rate of 10 °C/min in 100 ml/min Ar. During the heating, the effluent gas was recorded on an online MS for analysis.

4. Computational method

Density functional theory calculations were performed within the framework of the first-principles method by the Vienna Ab-initio Simulation Package (VASP) [1]. Interactions between electrons and nuclei were described by the projector augmented wave (PAW) pseudopotentials, while the exchange-correlation interactions were described by the GGA-PBE function [2]. The Kohm-Sham valence states were expanded in a plane wave basis set with the kinetic energy cutoff of 450 eV. The energy and ion force convergence thresholds were 10^{-5}eV and 0.03 eV/Å^2. The model structure used in this simulation was based on three carbon layers with a 6 × 6 supercell, and a vacuum space of 12 Å in the z-direction. A 2*2*1 k-point sampling was used in the Brillouin zone integration. To model the structure, carbon vacancy was created to host the doping elements. Transition states were searched by the climbing image nudged-elastic-band (CI-NEB) method with a convergence criterion of 0.05 eV/Å^2 [3]. Vibrational analyses were also performed to verify the transition state and local minimum. The activation energy barrier E_a and the reaction energy ΔE_r were determined as

$$E_a = E_{TS} - E_{IS};\ \Delta E_r = E_{FS} - E_{IS}$$

where E_{TS}, E_{IS}, and E_{FS}, are the energy of the transition state, the initial state, and the final state.

S2. Results and discussions

The reactions involved in ethylene oxychlorination (Equation 1) and the redox cycles (Equations 2−4).

Total reaction:	$C_2H_4 + 0.5O_2 + 2HCl \rightarrow C_2H_4Cl_2 + H_2O$	(1)
Reduction:	$2CuCl_2 + C_2H_4 \rightarrow C_2H_4Cl_2 + 2CuCl$	(2)
Oxidation:	$2CuCl + 0.5O_2 \rightarrow Cu_2OCl_2$	(3)
Hydrochlorination:	$Cu_2OCl_2 + 2HCl \rightarrow 2CuCl_2 + H_2O$	(4)

Table S1. BET surface area, pore volume, and pore size of the catalysts.

	Surface area (m^2/g)	Pore volume (cm^3/g)	Pore size (nm)
NPC	2059	0.92	2.78
Cu-NC	2060	0.97	2.78
Cu-NPC	2070	1.00	2.81
Cu-NPC spent	2122	1.01	2.80
Cu-NC spent	2046	0.96	2.76

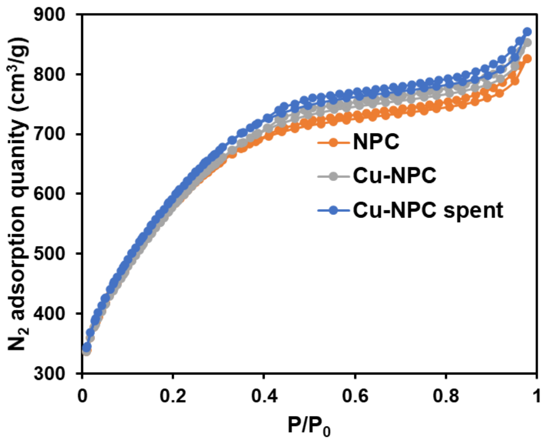

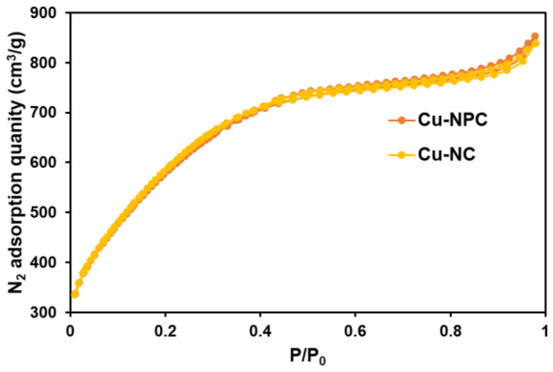

Figure S1. N_2 adsorption-desorption isotherms of the fresh and spent Cu-NPC and NPC catalysts.

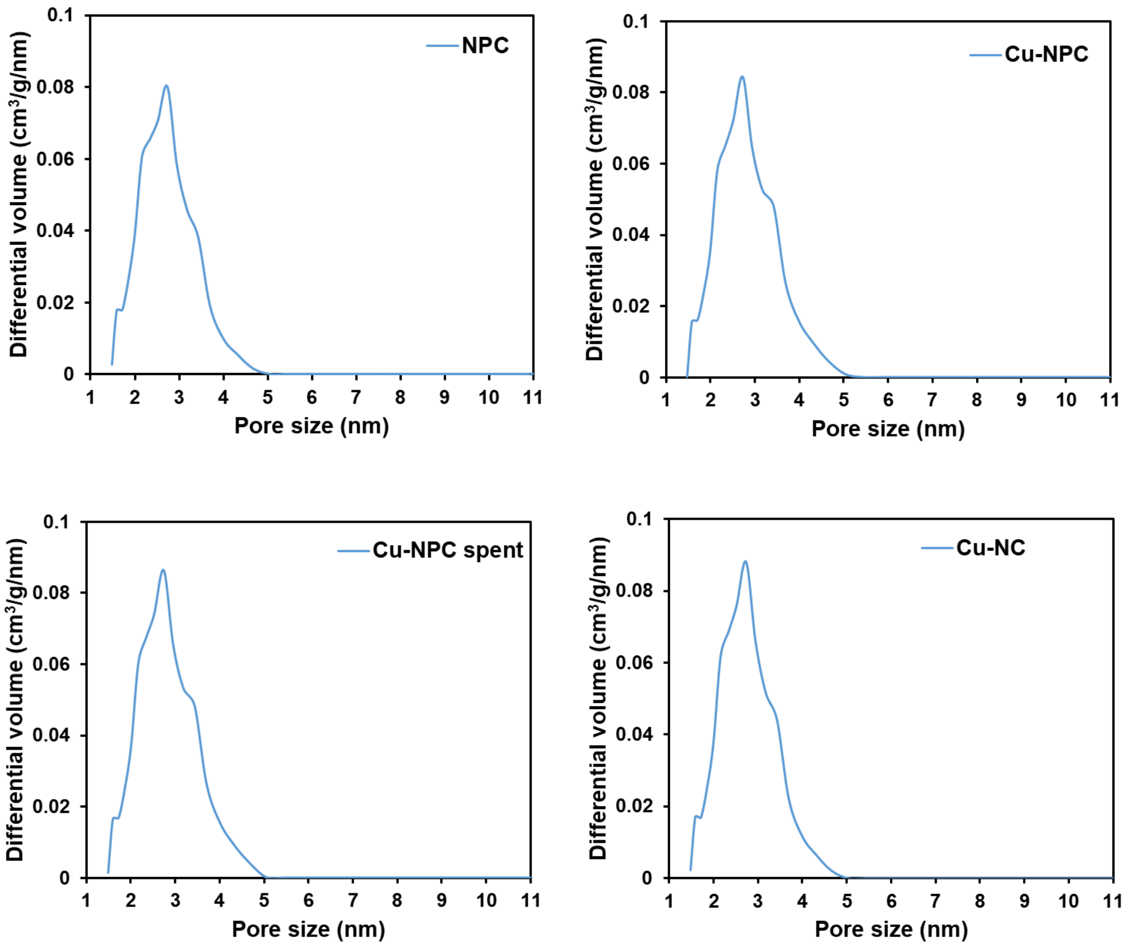

Figure S2. The pore size distribution of the catalysts, calculated by the DFT method.

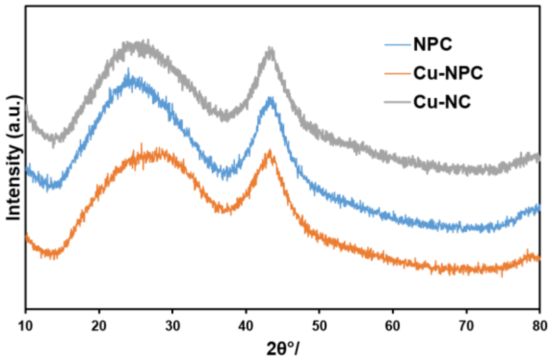

Figure S3. XRD patterns of the fresh catalysts.

Table S2. Element's concentration of the samples (Note: Cu and P are obtained from ICP, N is obtained from XPS).

Element loading (wt %)	Cu	N	P
Cu-NPC	0.24	1.63	0.1
Cu-NC	0.28	1.65	-

Table S3. Cu K-edge EXAFS fitting parameters of Cu-NPC. (Notes: N, coordination number; R, the internal atomic distance; ΔE_0, the edge-energy shift; σ^2, Debye-Waller factor.)

Path	N	R(Å)	ΔE_0 (eV)	σ^2 (10^{-3}Å^2)	R factor
Cu−N	3.5 ± 0.1	1.95 ± 0.01	−2.7 ± 0.4	5.5 ± 0.6	0.002
Cu−P	0.4 ± 0.1	2.39 ± 0.02	7.2 ± 1.9	3.4 ± 2.6	

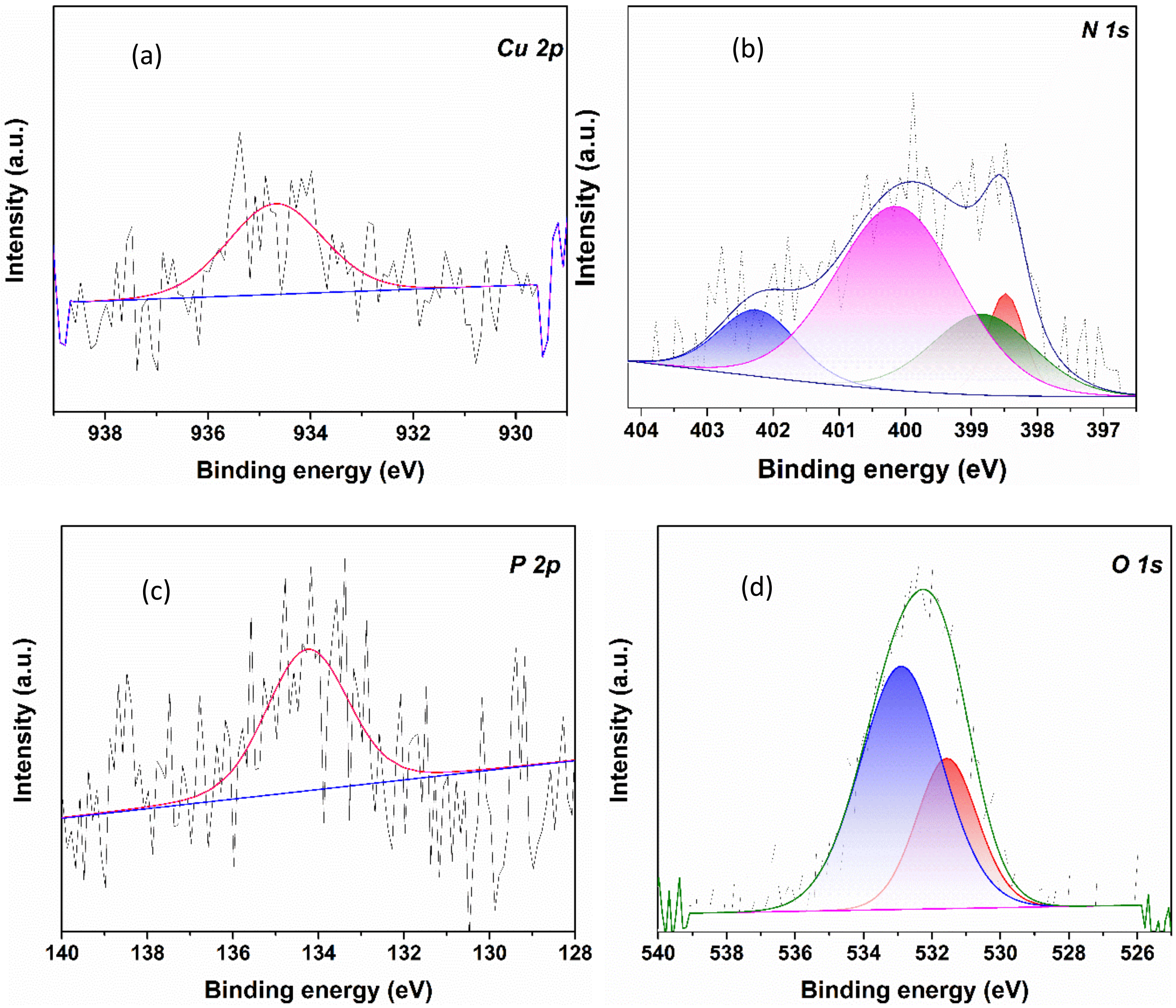

Figure S4. XPS spectra of Cu-NPC. (a) Cu 2p, (b) N 1s, (c) P 2p, and (D) O 1s.

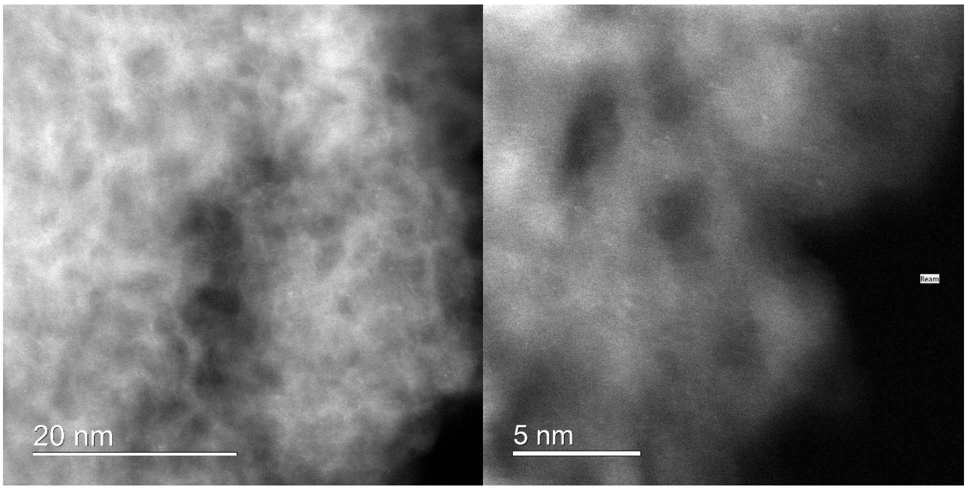

Figure S5. HAADF-STEM images of Cu-NPC catalyst.

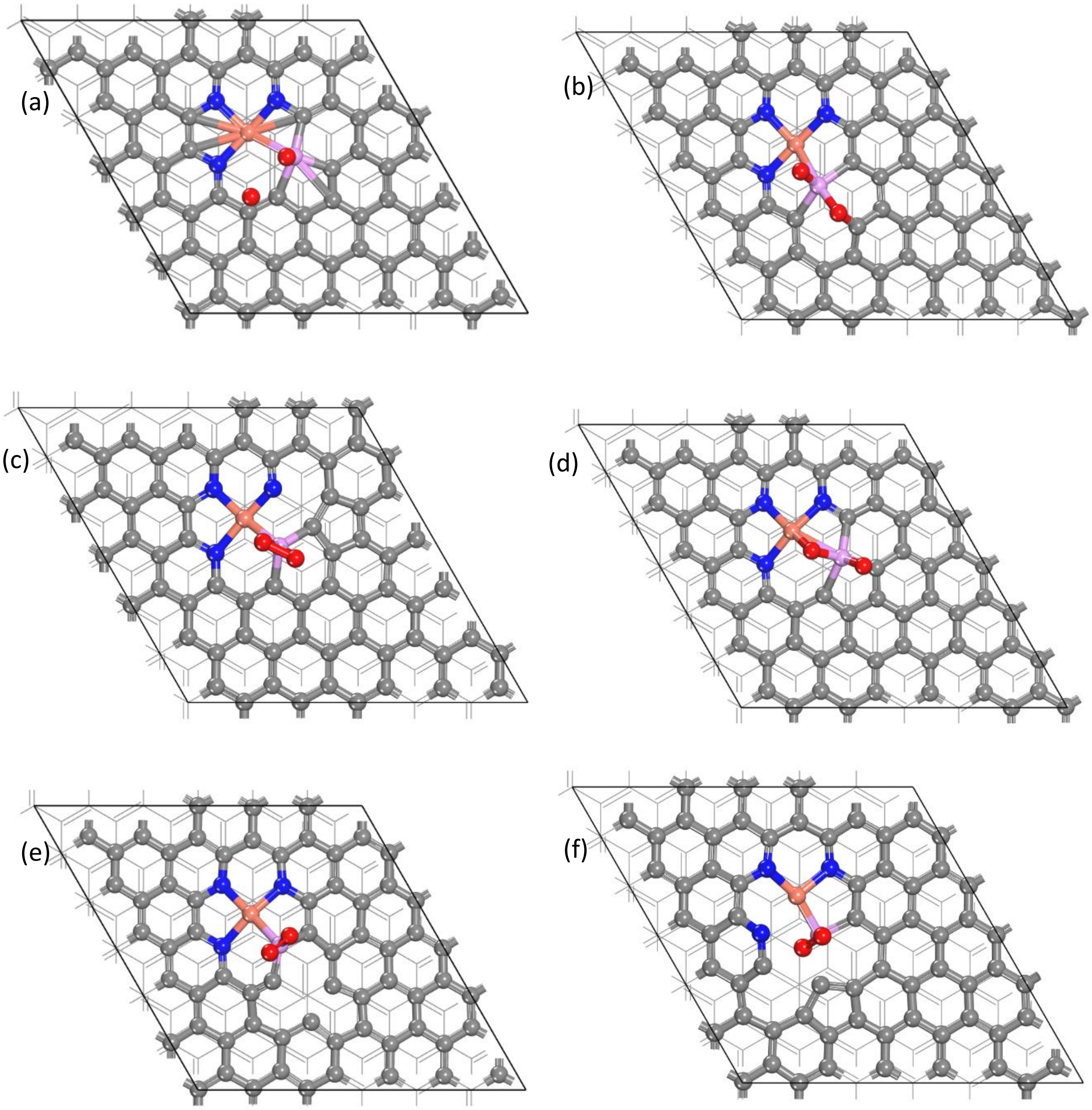

Figure S6. Schematic model of the possible Cu-NPC catalyst (grey: carbon; red: oxygen; blue: nitrogen; the light shade of red: Cu; light magenta: phosphorus).

The final energy of the proposed structure is (a) 40.75eV, (b) -7.34eV, (c) -1.82eV, (d) -6.37eV, (e) 0.96eV, (f) 224.16eV. Therefore, the schematic of b has the most stable structure.

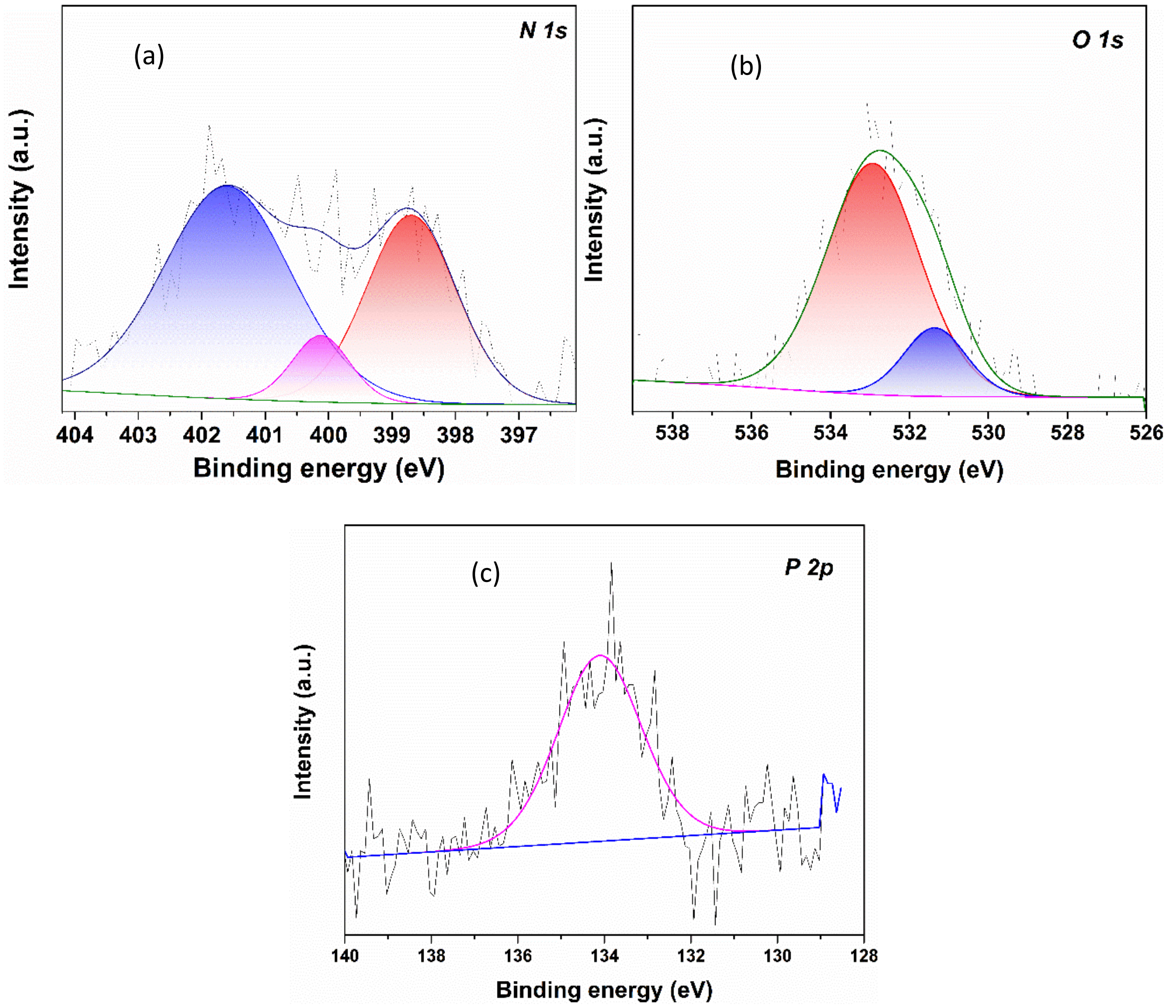

Figure S7. XPS spectra of (a) N 1s, (b) O 1s, (c) P 2p of the NPC.

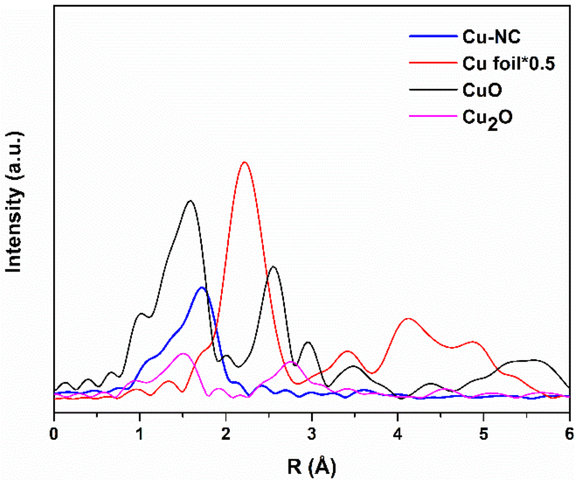

Figure S8. Fourier transform-EXAFS spectra of Cu-NC and the standards.

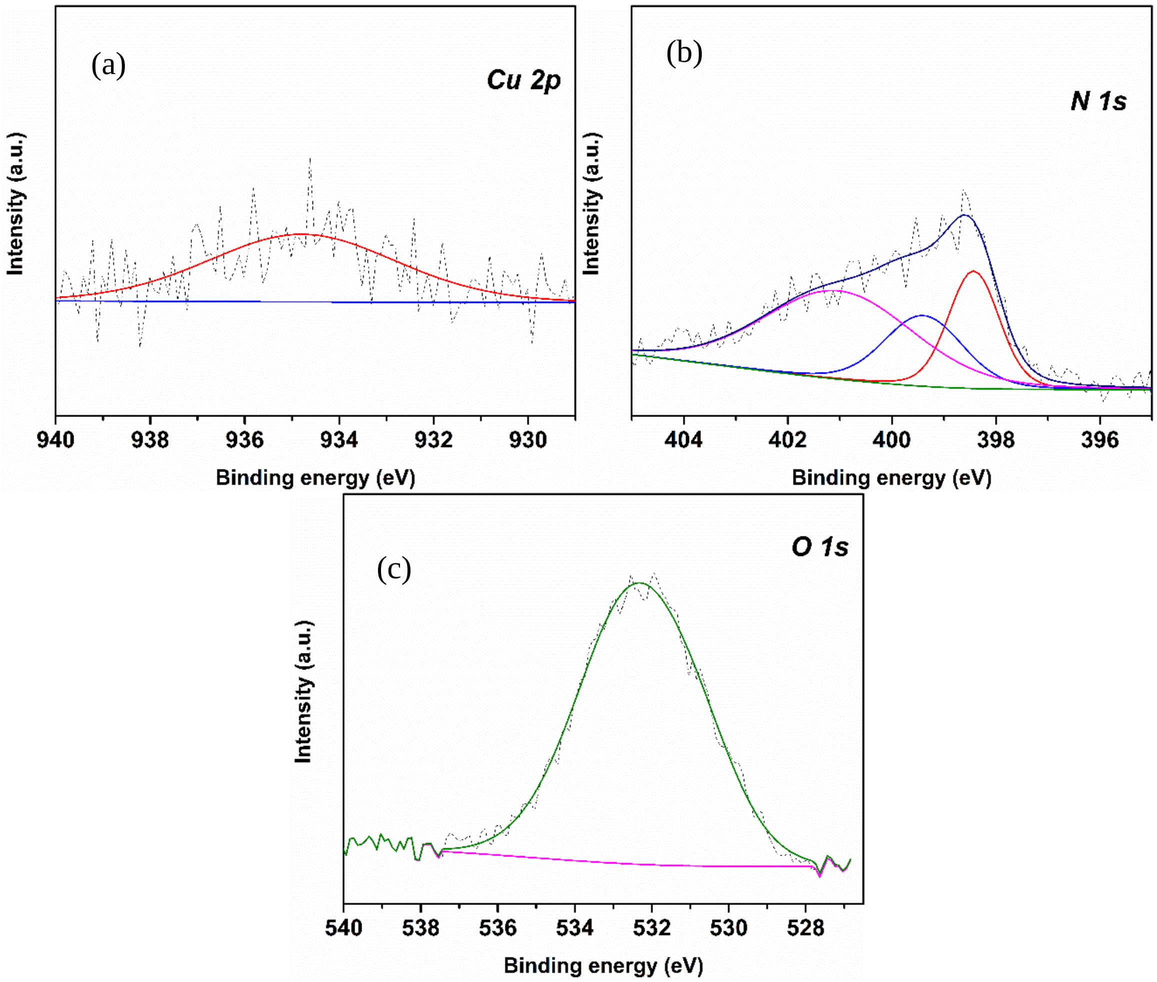

Figure S9. XPS spectra of (a) Cu 2p, (b) N 1s, and (c) O 1s of the Cu-NC catalyst.

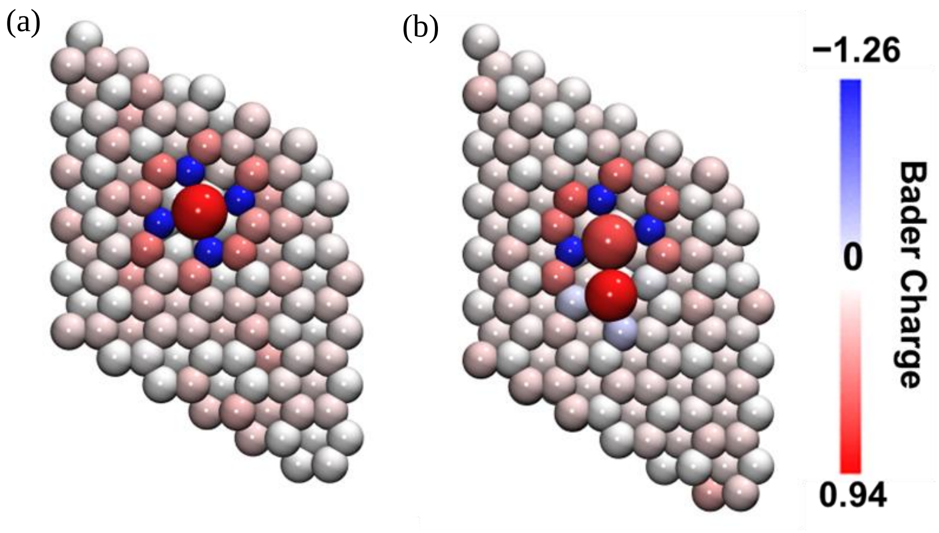

Figure S10. Bader charge population analysis of the (a) Cu-NC, and (b) Cu-NPC catalysts. Atoms color: blue, N; light red (in the central): Cu; red near the central atom: P.

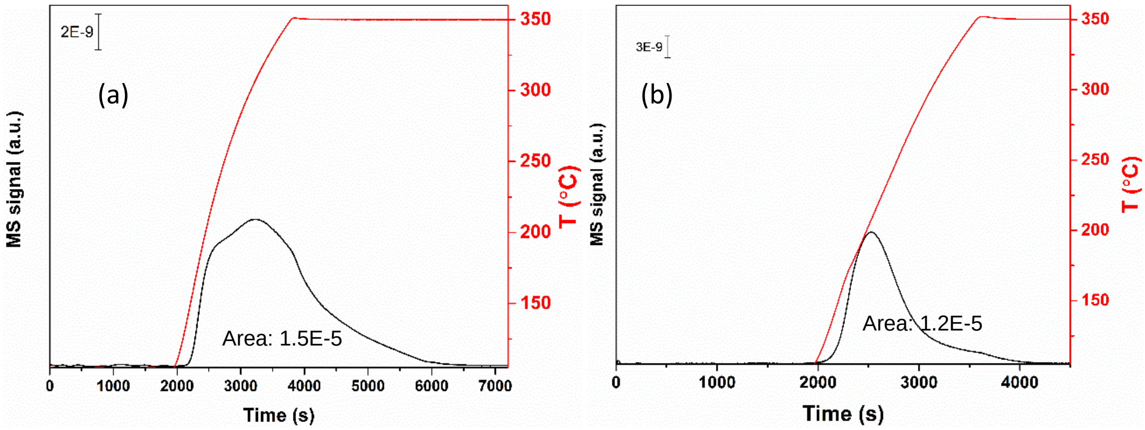

Figure S11. TPD profiles of the carbon catalysts. HCl evolution curves obtained from TPD analysis of the (a) Cu-NC and (b) Cu-NPC catalysts, Conditions: W_{cat}= 0.2 g, F_{Ar}= 100 ml/min, ramping rate 10 °C/min.

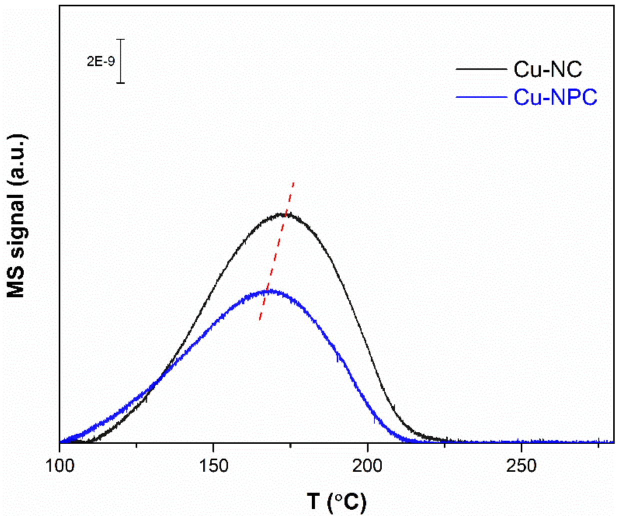

Figure S12. EDC temperature programmed desorption profiles over Cu-NC and Cu-NPC catalysts. Conditions: W_{cat} = 0.2 g, F_{Ar} = 100 ml/min, 10 °C/min.

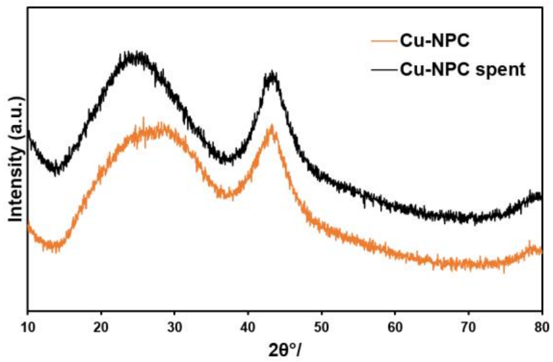

Figure S13. XRD patterns of the fresh and spent Cu SAC catalysts.

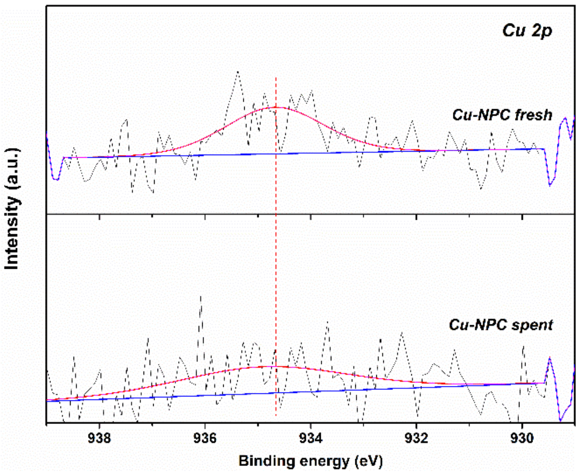

Figure S14. Cu 2p XPS spectra of the fresh and spent Cu-NPC catalysts.

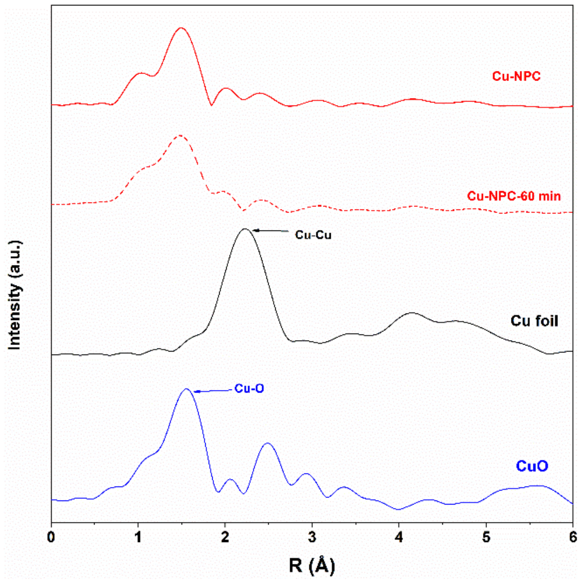

Figure S15. EXAFS spectra of Cu-NPC before and after reaction, and Cu foil, CuO standards.

Table S4. Conversion and product selectivity of the NPC catalyst (without Cu). Reaction conditions: W_{cat} = 0.2 g, T = 250 °C, P_{total} = 1 bar, C_2H_4/20%O_2/20%HCl/N_2: 4/10/40/4 ml/min.

	**Selectivity/%**							
**Conv./%**	CO_2	VCM	EDC	C_2H_5Cl	1,1-$C_2H_4Cl_2$	1,2-$C_2H_2Cl_2$	CCl_4	1,1,2-$C_2H_3Cl_3$
2.0	31.4	25.4	18.5	10.1	3.8	5.2	5.0	0.6

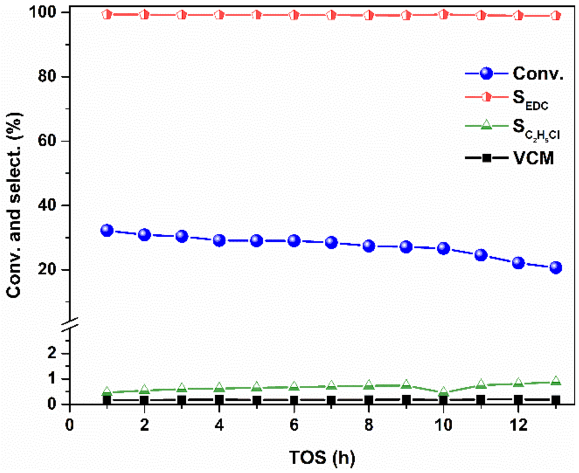

Figure S16. Conversion and product selectivity of the CeCu/Al_2O_3 catalyst (with 5wt%Cu). Reaction conditions: W_{cat} = 0.2 g, T = 250 °C, P_{total} = 1 bar, flow rate (ml/min): C_2H_4/20%O_2/20%HCl/N_2: 4/10/40/4.

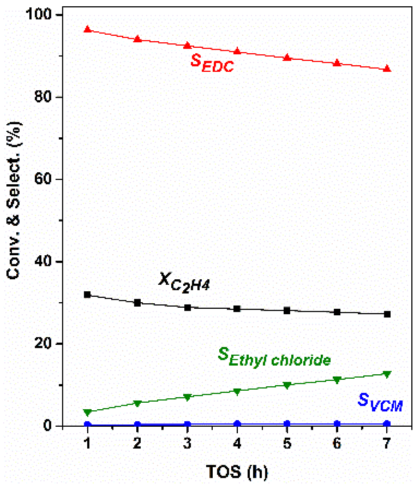

Figure S17. Conversion and product selectivity of the Cu/Al_2O_3 catalyst (with 5wt%Cu). Reaction conditions: W_{cat} = 0.2 g, T = 250 °C, P_{total} = 1 bar, C_2H_4/20%O_2/20%HCl/N_2: 4/10/40/4 ml/min.

Table S5. Catalytic performance comparison with the reported Cu/Al_2O_3-based catalysts.

Catalyst	Cu wt%	T (°C)	C_2H_4 conv. (%)	S_{EDC} (%)	Production rate ($mol/g_{cat}/h$)	Ref.
$CuCl_2$/ γ-Al_2O_3	5	230	10	98	5.25E-03	[4]
CeLa-$CuCl_2$/Al_2O_3	5	230	15	99	7.96E-3	
$CuCl_2$/ γ-Al_2O_3	5	250	17.4	85.2	4.77E-03	[5]
$CuCl_2$/TiO_2	5	250	13	95	3.97E-03	
$CuCl_2$/H-Beta25	5	250	1.3	10.1	4.2E-05	
$CuCl_2$/SiO_2	5	250	0.3	92.2	8.89E-05	
Cu-NPC	0.24	250	37	98.1	1.96E-2	Current work

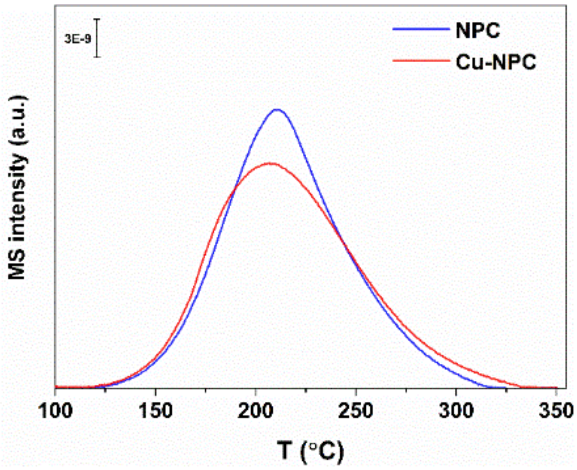

Figure S18. TPD profiles of the carbon catalysts. HCl evolution curves obtained from TPD analysis of the Cu-NPC and NPC catalysts, Conditions: W_{cat}= 0.2 g, F_{Ar}= 100 ml/min, ramping rate 10 °C/min.

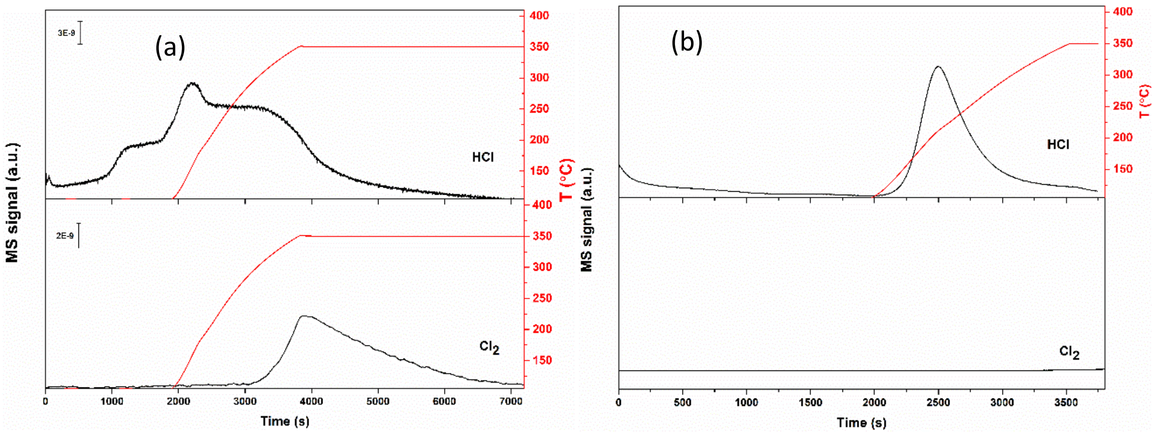

Figure S19. O_2-TPSR results. (a) Cu-NC, (b) NPC after the catalysts were saturable adsorbed HCl, with 10% O_2 flow to the reactor. Reaction conditions: W_{cat} = 0.2 g, F_{tot}= 100 ml/min, ramping rate 10 °C/min.

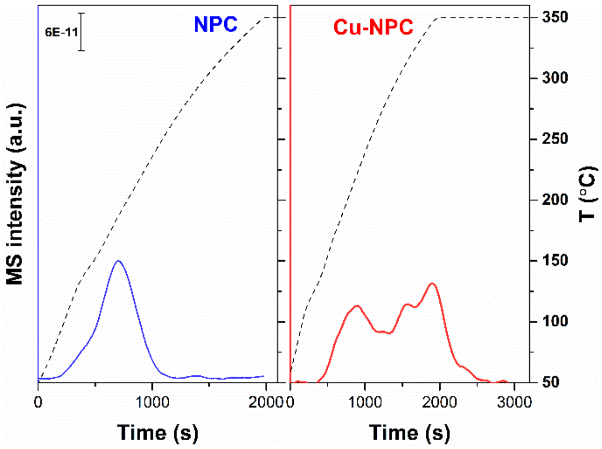

Figure S20. TPD profiles of the carbon catalysts. C_2H_4-TPD analysis of the NPC and Cu-NPC catalysts. Conditions: W_{cat}= 0.2 g, F_{Ar}= 100 ml/min, ramping rate 10 °C/min.

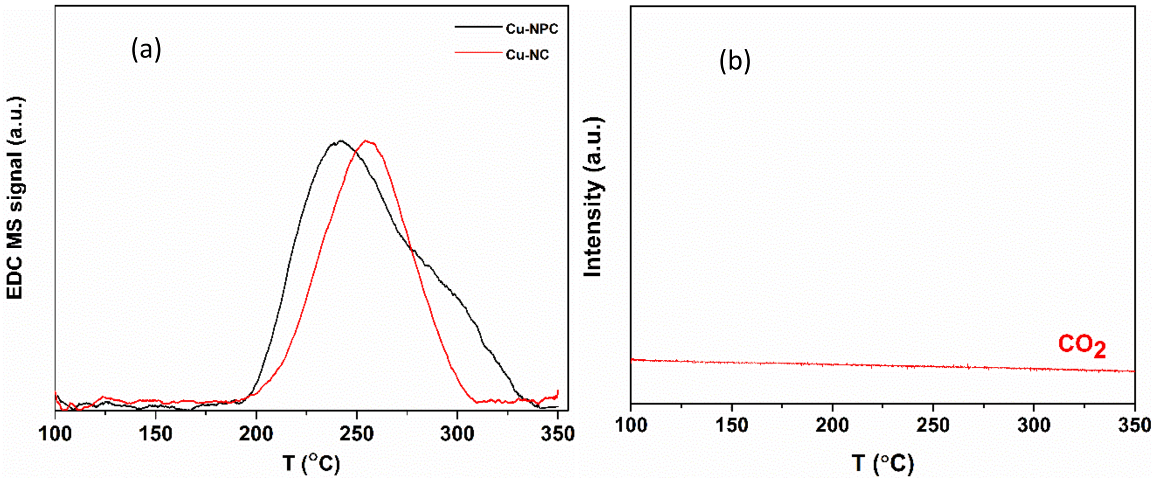

Figure S21. C_2H_4/O_2-TPSR results. (a) EDC evolution file on Cu-NPC and Cu-NC, (b) CO_2 evolution, after the catalysts were saturable adsorbed HCl, with C_2H_4/10% O_2/He=4/10/44 (ml/min) flow to the reactor. Reaction conditions: W_{cat} = 0.2 g, 10 °C/min.

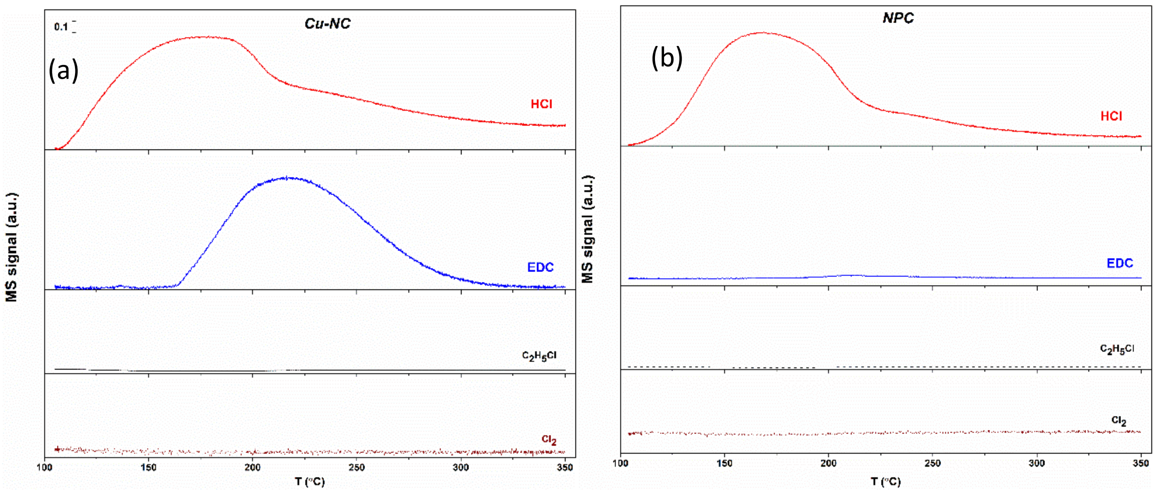

Figure S22. C_2H_4-TPSR results after the catalysts were saturable adsorbed HCl, with 10% C_2H_4 flow to the reactor. (a) Cu-NC, (b) NPC. Reaction conditions: W_{cat} = 0.2 g, F_{tot}= 100 ml/min, ramping rate 10 °C/min.

Table S6. Reaction pathways involved in the ethylene oxychlorination on the Cu-NPC catalyst (* represents the active site).

O_2 (g) + * = O_2*	R1
O_2* + * = 2O*	R2
HCl (g) + * = HCl*	R3
HCl* + O* = Cl* +OH*	R4
C_2H_4 (g) + * = C_2H_4*	R5
C_2H_4* + Cl* = C_2H_4Cl* +*	R6
C_2H_4Cl* +Cl* = $C_2H_4Cl_2$* +*	R7
$C_2H_4Cl_2$* = $C_2H_4Cl_2$ (g) + *	R8
2OH* = H_2O* + O*	R9
H_2O* =H_2O (g) + *	R10

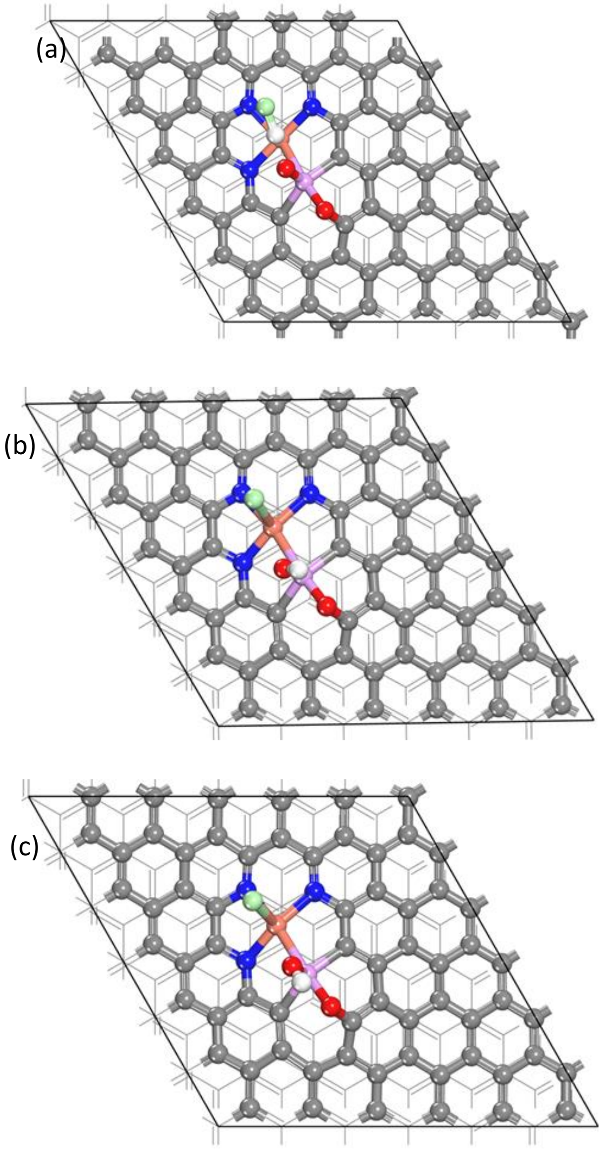

$$O * + \mathrm{HCl} * \rightarrow \mathrm{Cl} * + \mathrm{OH} *$$

Figure S23. The schematic model structure over the Cu-NPC catalyst in the HCl oxidative dissociation step. (a) O*+HCl*, (b) transition state, (c) Cl*+OH*. (Grey: carbon; red: oxygen; blue: nitrogen; the light shade of red: Cu; light magenta: phosphorus)

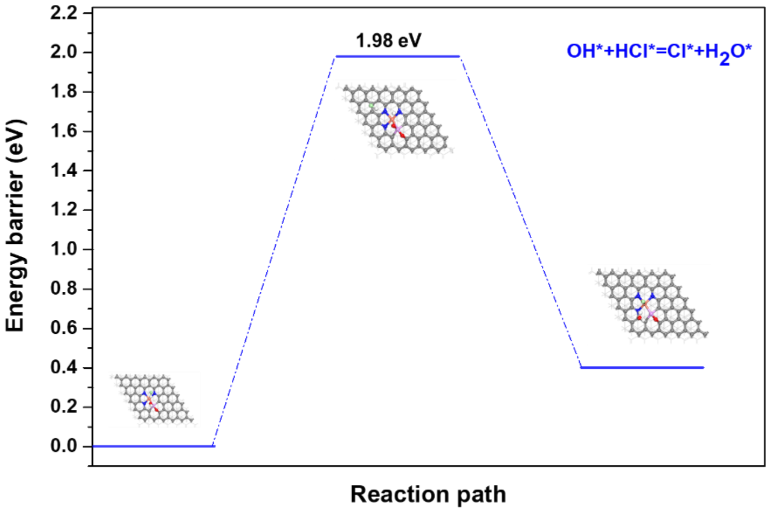

Figure S24. Energy diagram of the HCl oxidative dissociation with OH on the Cu-NPC catalyst.

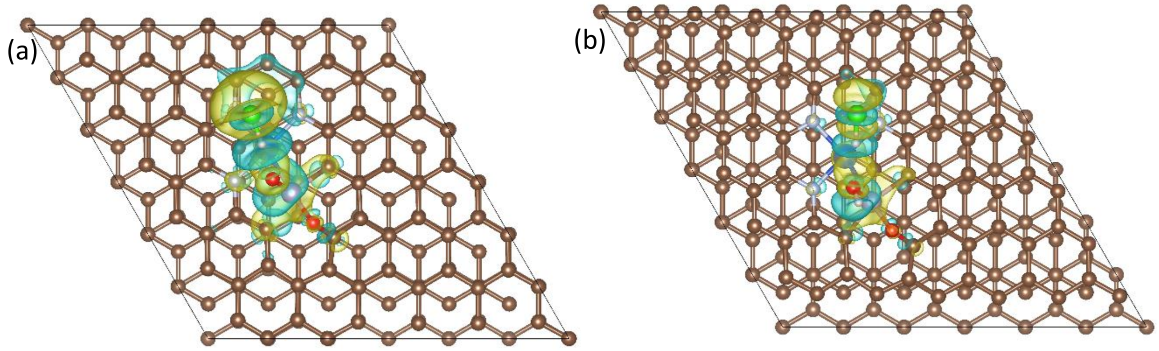

Figure S25. Difference of charge density for the HCl* adsorption configurations on (a) O*, and (b) OH*. The yellow region represents charge accumulation, and the charge depletion is in the blue region. Atom colors, green: Cl, white: H, red: O, blue: Cu, brown: C, purple: P, silver: N.

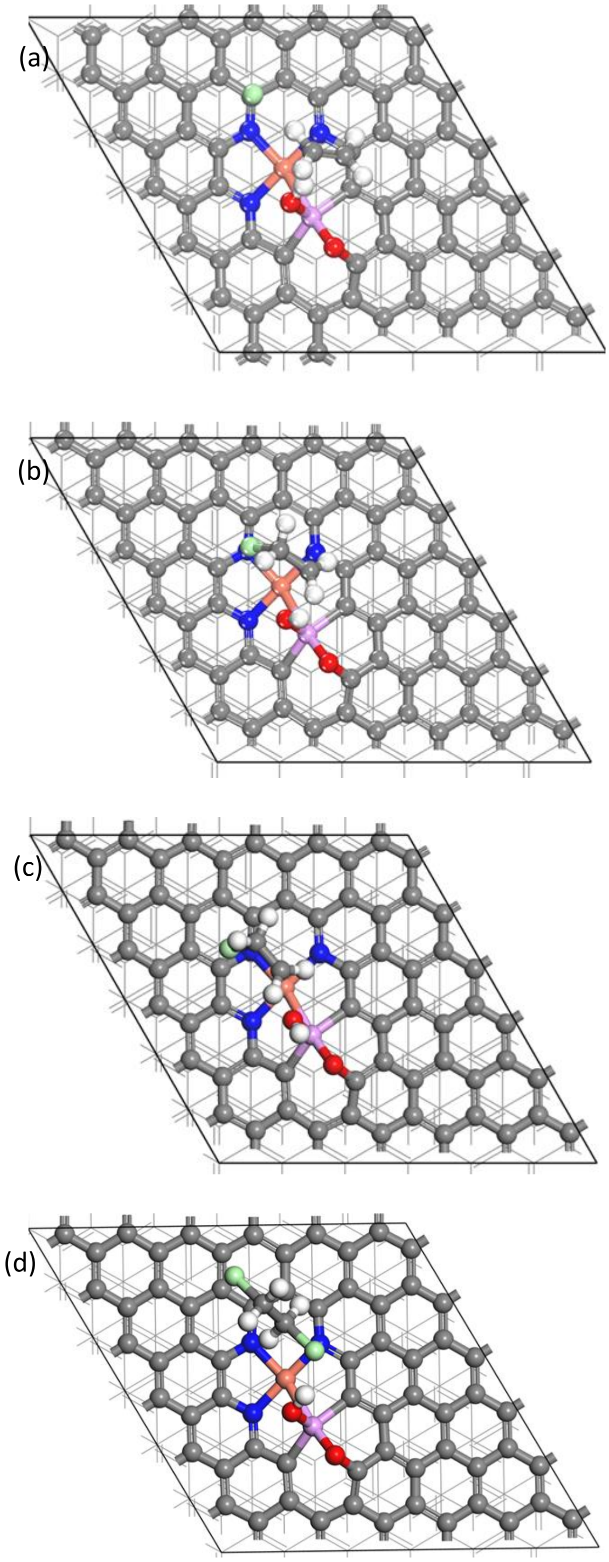

$$C_2H_4 + Cl * \rightarrow C_2H_4Cl * \rightarrow C_2H_4Cl_2 *$$

Figure S26. The schematic model structure over the Cu-NPC catalyst in the EDC formation step. (a) C_2H_4*+Cl*, (b) transition state, (c) C_2H_4Cl*, (d) $C_2H_4Cl_2$*. (Grey: carbon; red: oxygen; blue: nitrogen; the light shade of red: Cu; light magenta: phosphorus).

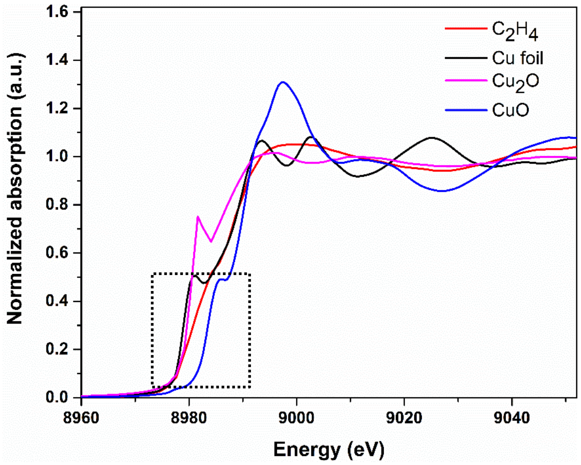

Figure S27. *In situ* Cu K-edge XANES spectra of Cu-NPC after the C_2H_4 reduction step Reaction condition: 250 °C, P_{C2H4}=0.069 bar, the total flow rate of 10 ml/min.

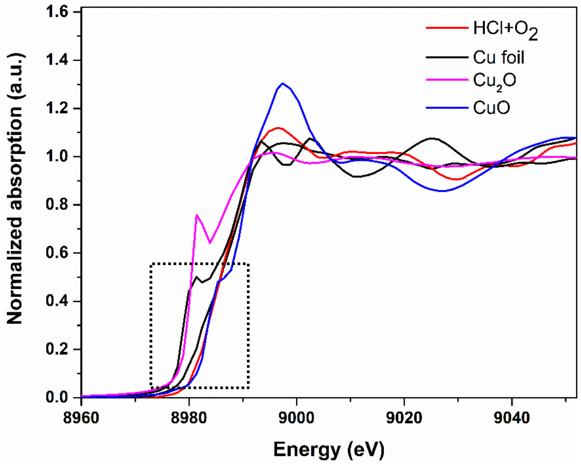

Figure S28. *In situ* Cu K-edge XANES spectra of Cu-NPC after the HCl+O_2 oxidation step Reaction condition: 250 °C, P_{HCl}=0.14 bar, O_2/HCl=1/4, the total flow rate of 10 ml/min.

Table S7. Frequency of different adsorption models of the transition state in the step of HCl dissociation with oxygen.

entry	ν (cm^{-1})
f1	3589.3
f2	872.0
f3	158.6
f4	116.2
f5	54.9
f6/i	309.3

Table S8. Frequency of different adsorption models of the transition state in the step of C_2H_4 chlorination. (Note: the imaginary frequencies less than 50i cm^{-1} will not be considered[6])

entry	ν (cm^{-1})
f1	3183.5
f2	3077.2
f3	3071.6
f4	3000.0
f5	1433.3
f6	1404.6
f7	1225.6
f8	1168.4
f9	1073.8
f10	1004.6
f11	763.3
f12	631.5
f13	541.8
f14	309.2
f15	232.7
f16	97.3
f17	54.4
f18	26.3
f19	13.5
f20/i	2.4
f21/i	53.1

References

[1] a) G. Kresse, J. Furthmüller, *Phys. Rev. B* **1996**, *54*, 11169; b) G. Kresse, J. Furthmüller, *Comput. Mater. Sci.* **1996**, *6*, 15-50.

[2] J. P. Perdew, K. Burke, M. Ernzerhof, *Phys. Rev. Lett.* **1996**, *77*, 3865.

[3] a) G. Mills, H. Jónsson, G. K. Schenter, *Surf. Sci.* **1995**, *324*, 305-337; b) G. Henkelman, B. P. Uberuaga, H. Jónsson, *J. Chem. Phys.* **2000**, *113*, 9901-9904.

[4] N. B. Muddada, T. Fuglerud, C. Lamberti, U. Olsbye, *Top. Catal.* **2014**, *57*, 741-756.

[5] Z. Vajglova, N. Kumar, K. Eranen, A. Tokarev, M. Peurla, J. Peltonen, D. Y. Murzin, T. Salmi, *Appl. Catal. A* **2018**, *556*, 41-51.

[6] H. Wang, Y. Xie, R. B. King, H. F. Schaefer Iii, *Inorg. Chem.* **2008**, *47*, 3045-3055.
